# Circulating cell adhesion molecules as biomarkers in inflammatory bowel disease: a systematic review and meta-analysis

**DOI:** 10.3389/fimmu.2025.1680317

**Published:** 2025-12-01

**Authors:** Krzysztof Przęczek, Dorota Cibor, Małgorzata Zwolińska-Wcisło, Danuta Owczarek

**Affiliations:** 1 Department of Gastroenterology and Hepatology, Faculty of Medicine, Jagiellonian University Medical College, Krakow, Poland; 2 Doctoral School of Medical and Health Sciences, Jagiellonian University Medical College, Krakow, Poland

**Keywords:** biomarkers, cell adhesion molecules, crohn’s disease, inflammatory bowel disease, immunoglobulin-like cell adhesion molecules, endothelial dysfunction, ulcerative colitis, selectins

## Abstract

**Introduction:**

Endothelial dysfunction is a recognized component of the pathogenesis and clinical course of inflammatory bowel disease (IBD). Measurement of soluble forms of cell adhesion molecules (CAMs) may reflect the extent of endothelial injury and serve as potential biomarkers of disease activity. We conducted a systematic review and meta-analysis of studies reporting soluble intercellular adhesion molecule−1 (sICAM−1), vascular cell adhesion molecule−1 (sVCAM−1), mucosal addressin cell adhesion molecule−1 (sMAdCAM−1), and selectins (sE−selectin, sP−selectin, and sL−selectin) in patients with IBD and healthy controls, or in comparable IBD subgroups defined by disease activity or type.

**Methods:**

We systematically searched PubMed, Embase, Web of Science, and Scopus from inception to June 15, 2025. Risk of bias was assessed using a modified Newcastle–Ottawa Scale.

**Results:**

Twenty-six studies met the inclusion criteria. Compared with healthy controls, patients with IBD showed higher levels of sICAM-1 (SMD 1.38, 95% CI 0.51 to 2.25, p=0.002) and sE-selectin (SMD 0.35, 95% CI 0.09 to 0.61, p=0.008). In subgroup analyses, this association persisted for sICAM-1 in both Crohn’s disease (CD) (SMD 1.89, 95% CI 0.15 to 3.62, p=0.033) and ulcerative colitis (UC) (SMD 0.95, 95% CI 0.25 to 1.64, p=0.008), and for sE-selectin only in CD (SMD 0.43, 95% CI 0.04 to 0.82, p=0.032). When comparing active and inactive disease, higher sICAM-1 levels were observed in the active group (SMD 0.75, 95% CI 0.38 to 1.12, p<0.001), while no significant differences were found for other CAMs. No differences in levels of these molecules were observed between CD and UC.

**Conclusions:**

Circulating CAMs, particularly sICAM-1 and sE-selectin, are elevated in IBD patients, supporting a role of endothelial injury in disease pathogenesis. Among these, sICAM-1 shows potential as a biomarker for distinguishing active from inactive disease.

**Systematic Review Registration:**

https://www.crd.york.ac.uk/PROSPERO/, identifier CRD420251088622.

## 1 Introduction

Inflammatory bowel disease (IBD) comprises a group of chronic gastrointestinal disorders characterized by alternating periods of exacerbation and remission. The two primary subtypes are ulcerative colitis (UC), which is typically confined to the colon and manifests clinically with diarrhea and rectal bleeding, and Crohn’s disease (CD), which can affect any segment of the gastrointestinal tract and may lead to complications such as fistulas, strictures, and abscesses (1). Importantly, both during active phases and remission, IBD may be associated with a range of extraintestinal manifestations, including malnutrition, anemia, arthritis, dermatologic lesions, hepatobiliary disorders, and cardiovascular complications, notably venous thromboembolic events (2–6). IBD occurs more frequently in industrialized countries; however, epidemiological data indicate a global increase in incidence, affecting both developed and developing regions worldwide (7). The pathogenesis of IBD is multifactorial and not yet fully understood. It involves genetic susceptibility, environmental factors, disturbances in the gut microbiota, immune dysregulation, and endothelial dysfunction (ED) (1, 8, 9). The vascular endothelium plays a crucial role in multiple physiological processes, including the regulation of coagulation, cell proliferation and angiogenesis, leukocyte migration, and the inflammatory response. ED, primarily associated with a decrease in production or activity of the vasodilator nitric oxide (NO), leads to increased expression of cell adhesion molecules (CAMs), elevated vascular wall permeability, and augmented leukocyte transmigration (10–13).

The main groups of CAMs include integrins, selectins, and the immunoglobulin superfamily of CAMs, which encompasses, e.g., intercellular adhesion molecule-1 (ICAM-1), vascular cell adhesion molecule-1 (VCAM-1), and mucosal addressin cell adhesion molecule-1 (MAdCAM-1) (14). Selectins exist in three forms: L-selectin, expressed on leukocytes; E-selectin, expressed on activated endothelial cells; and P-selectin, found on activated platelets and endothelial cells. Selectins participate in the initial phase of the adhesion cascade by mediating the rolling of leukocytes along the vascular endothelium (15, 16). During inflammatory conditions, the expression of ICAM-1 is upregulated, and its primary ligands are leukocyte-specific β2 integrins, CD11 and CD18. Similarly, VCAM-1 is upregulated and interacts with the α4β1 integrin, which is expressed on leukocytes (17, 18). MAdCAM-1 is predominantly expressed on activated endothelial cells in the intestinal mucosa and interacts with the α4β7 integrin present on the surface of lymphocytes (19, 20). Soluble forms of the aforementioned adhesion molecules serve as biomarkers of endothelial activation and function in various diseases, including IBD (17, 21–24).

To evaluate the utility of selected soluble CAMs as disease biomarkers, we conducted a systematic review and meta-analysis of studies assessing their serum and plasma concentrations in patients with IBD compared to healthy controls, with primary outcomes focused on differences between IBD patients and controls, and secondary outcomes including comparisons between CD and UC as well as between active and inactive disease states.

## 2 Materials and methods

This study was conducted in accordance with the Preferred Reporting Items for Systematic Reviews and Meta-Analyses (PRISMA) guidelines (25). The study protocol was registered in the International Prospective Register of Systematic Reviews (PROSPERO registration number: CRD420251088622).

### 2.1 Search strategy

We systematically searched PubMed, Embase, Web of Science, and Scopus from their inception to June 15, 2025, using the following search terms: (“ICAM” OR “Intercellular Adhesion Molecule” OR “sICAM” OR “VCAM” OR “sVCAM” OR “Vascular cell adhesion molecule” OR “Mucosal vascular addressin cell adhesion molecule” OR “MAdCAM” OR “sMAdCAM” OR “selectin” OR “L-selectin” OR “sL-selectin” OR “P-selectin” OR “sP-selectin” OR “E-selectin” OR “sE-selectin” OR “CD62L” OR “CD62P” OR “CD62E” OR “sCD62L” OR “sCD62P” OR “sCD62E”) AND (“Crohn” OR “ulcerative colitis” OR “inflammatory bowel disease” OR “IBD” OR “Crohn’s disease”). In addition, the reference lists of the included studies were manually reviewed to identify any additional eligible publications. Duplicate records were removed using the Bramer method in EndNote (26).

### 2.2 Eligibility criteria

The inclusion criteria were as follows: (1) measurement of serum or plasma concentrations of soluble forms of ICAM-1, VCAM-1, MAdCAM-1, E-selectin, P-selectin, or L-selectin; (2) a case–control design including either a healthy control group and IBD patients, a comparison between CD and UC patients, or a comparison between active and inactive IBD patients; (3) participants aged over 18 years; (4) study groups comprising at least five individuals; (5) publication written in English; and (6) full-text availability. Studies were excluded if they met any of the following criteria: (1) review articles, letters to the editor, case reports, or conference abstracts; (2) inclusion of patients with other inflammatory, ischemic, or neoplastic bowel diseases; or (3) duplicate publications or insufficient data for analysis.

### 2.3 Data extraction

Each abstract was independently screened by two reviewers (KP and DC). If considered potentially relevant, the full-text article was retrieved and evaluated for further assessment. Any disagreements between reviewers were resolved by a third reviewer (DO). From the included studies, the following data were extracted: country of study, year of publication, participant age, male-to-female ratio, biomarker analyzed with units, assay method, IBD subtype, disease activity, disease phenotype, and treatment details. When data were not reported in numerical form, values were extracted from graphs using WebPlotDigitizer (version 5.2; Ankit Rohatgi, CA, USA). Medians, interquartile ranges (IQRs), and complete ranges were converted to means and standard deviations using validated statistical methods (27, 28).

### 2.4 Bias assessment

Risk of bias was assessed using a modified Newcastle-Ottawa Scale (NOS) for case-control studies. Since the non-response rate was not applicable, the maximum attainable score was 8 points (29, 30). Studies were categorized based on their NOS scores as follows: 0–3 points indicated low quality with high risk of bias, 4–6 points indicated moderate quality and risk, and 7–8 points indicated high quality with low risk of bias. In studies lacking a healthy control group, comparisons were made between the groups being studied, such as CD vs UC or inactive vs active disease.

### 2.5 Statistical analysis

The primary outcome was the difference in biomarkers concentrations between patients with IBD and healthy controls, with subgroup analyses conducted separately for CD and UC. Secondary outcomes included comparisons between CD and UC, as well as between active and inactive IBD. Heterogeneity between studies was assessed using Cochran’s Q test, with a significance level set at p<0.10 (31). The degree of heterogeneity was quantified using the I² statistic, and categorized as low (<25%), moderate (25–75%), or high (>75%) (32, 33). Due to substantial heterogeneity observed across most studies, a random-effects model was applied to calculate standardized mean differences (SMDs) along with 95% confidence intervals (CIs) (34). Hedges’ correction was used to adjust for small-sample bias. The effect size was considered small for SMD<0.5, moderate for SMD between 0.5 and 0.8, and large for SMD>0.8. A p-value of <0.05 was considered statistically significant (33). Forest plots were generated from the calculated SMDs to visualize effect sizes across studies. Sensitivity analysis was performed by sequentially removing each study to assess its influence on the overall effect size. Publication bias was evaluated using Begg’s rank correlation test and Egger’s regression asymmetry test, and visually assessed using funnel plots, when at least ten studies were included (35, 36). When publication bias was suggested, the Duval and Tweedie “trim-and-fill” method was applied to estimate the potential impact of missing studies (37). Subgroup analyses were performed for biomarkers reported in at least five studies to investigate potential differences in effect sizes across disease types and disease activity levels. Meta-regression was not performed due to the limited number of included studies and the insufficient reporting of demographic and clinical data. Statistical analyses were performed using STATA version 19 (StataCorp LLC, College Station, TX, USA).

## 3 Results

### 3.1 Study selection

**Figure 1:**
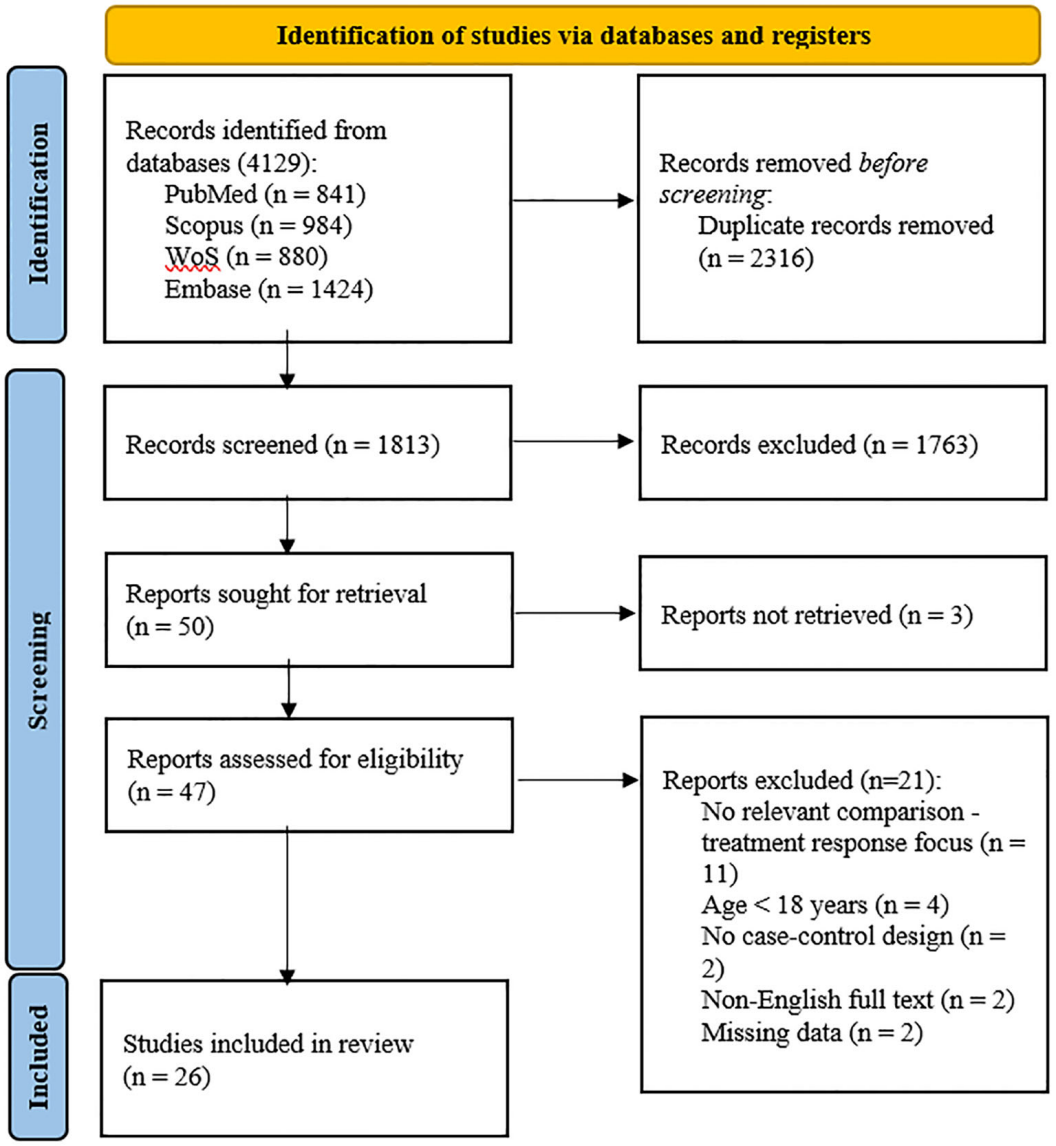
PRISMA 2020 flow diagram.

A total of 4,129 records were identified; 2,316 duplicates were removed, and 1,763 records were excluded after screening titles and abstracts. Among the remaining 50 articles, three could not be retrieved, eleven did not include relevant comparisons, four were conducted in populations under 18 years old, two lacked a case–control design, two were not available in English, and two had missing data. A total of 26 studies (13, 38–62) were included in the meta-analysis (**Table 1**). The flow chart of the selection process is presented in **Figure 1**. The risk of bias was rated as low in thirteen studies (13, 39, 40, 44, 45, 47, 50, 52, 53, 57, 58, 61, 62), moderate in twelve (41–43, 46, 48, 49, 51, 54–56, 59, 60), and high in one study (38) (**Table 2**).

**Table 1:** Main characteristics of the studies included in the meta-analysis.

Study (subgroup)			Controls			IBD				
	Biomarker	Unit	n	M/F ratio	Age, years (mean ± SD)	n	M/F ratio	Age, years (mean ± SD)	IBD type	Activity assessment
Dippold W et al., 1993 (38), Germany	sICAM-1	ng/ml	19	NR	NR	25	NR	NR	CD	CDAI
Vainer B et al, 1994 (39), Denmark (CD)	E-selectin	nM	15	3/12	46.5 ± 6.9	15	6/9	42.3 ± 11.8	CD	Tvede
Vainer B et al, 1994 (39), Denmark (UC) (39)	E-selectin	nM	15	3/12	46.5 ± 6.9	16	5/11	41.7 ± 16.1	UC	Tvede
Nielsen OH et al., 1994 (40), Denmark (CD)	ICAM-1	ng/ml	29	9/20	40.3 ± 9.1	31	14/17	40 ± 11.2	CD	CDAI
Nielsen OH et al., 1994 (40), Denmark (UC)	ICAM-1	ng/ml	29	9/20	40.3 ± 9.1	27	14/13	40.8 ± 14.8	UC	Tvede
Jones SC et al., 1995 (41), England (CD)	ICAM-1, VCAM-1, E-selectin	ng/ml U/ml U/ml	27 90 90	NR	range 18-60	22	NR	range 19-68	CD	CDAI
Jones SC et al., 1995 (41), England (UC)	ICAM-1, VCAM-1, E-selectin	ng/ml U/ml U/ml	27 90 90	NR	range 18-60	21	NR	range 18-80	UC	Truelove and Witts
Patel RT et al., 1995 (42), England (CD)	ICAM-1 VCAM-1 E-selectin	ng/ml OD ng/ml	24	NR	32.5 ± 5.1	34	18/16	34.5 ± 10	CD	CDAI
Patel RT et al., 1995 (42), England (UC)	ICAM-1 VCAM-1 E-selectin	ng/ml OD ng/ml	24	NR	32.5 ± 5.1	49	NR	NR	UC	Sutherland
Göke M et al., 1997 (43), Germany (CD)	ICAM-1 VCAM-1 E-selectin P-selectin	ng/ml ng/ml ng/ml ng/ml	42 15 9 23	NR	NR	56 35 31 43	NR	NR	CD	CDAI
Göke M et al., 1997 (43), Germany (UC)	ICAM-1 VCAM-1 E-selectin P-selectin	ng/ml ng/ml ng/ml ng/ml	42 15 9 23	NR	NR	25 15 13 23	NR	NR	UC	Gomes
Bhatii M et al., 1998 (44), England (CD)	E-selectin	ng/ml	11	7/4	38 ± 15.7	16	9/7	42.8 ± 12.4	CD	CDAI
Bhatii M et al., 1998 (44), England (UC)	E-selectin	ng/ml	11	7/4	38 ± 15.7	16	8/8	43.8 ± 16.7	UC	Truelove and Witts
Vainer B et al., 1998 (45), Denmark	E-selectin	nM, ng/l	10	4/6	47.3 ± 6.8	30	13/17	43.8 ± 13.5	UC	Tvede
Seidelin JB et al., 1998 (46), Denmark (CD)	L-selectin	ng/ml	18	9/9	55.5 ± 13.7	16	6/10	42.3 ± 17.5	CD	Tvede
Seidelin JB et al., 1998 (46), Denmark (UC)	L-selectin	ng/ml	18	9/9	55.5 ± 13.7	23	13/10	40 ± 14.5	UC	Tvede
Fägerstam JP et al., 2000 (47), Sweden (CD)	P-selectin	ng/ml	12	6/6	46.5 ± 12.2	5	3/2	44 ± 14.4	CD	NR
Fägerstam JP et al., 2000 (47), Sweden (UC)	P-selectin	ng/ml	12	6/6	46.5 ± 12.2	16	10/6	46.8 ± 16	UC	NR
Goggins MG et al., 2001 (48), Ireland (CD)	ICAM-1 E-selectin	ng/ml ng/ml	18 14	NR	NR	38	12/26	40.8 ± 11	CD	HBI
Goggins MG et al., 2001 (48), Ireland (UC)	ICAM-1 E-selectin	ng/ml ng/ml	18 14	NR	NR	53	20/33	37.8 ± 9.5	UC	HBI
Vainer B et al., 2003 (49), Denmark	ICAM-1	ng/ml	10	0/10	45 ± 7.8	35	21/14	41.3 ± 14.9	UC	Truelove and Witts
Magro F et al., 2004 (50), Portugal (CD)	ICAM-1 VCAM-1 E-selectin P-selectin	ng/ml ng/ml ng/ml ng/ml	114	56/59	34.8 ± 7.7	145	61/84	39.5 ± 11	CD	HBI
Magro F et al., 2004 (50), Portugal (UC)	ICAM-1 VCAM-1 E-selectin P-selectin	ng/ml ng/ml ng/ml ng/ml	114	56/59	34.8 ± 7.7	73	30/43	37.8 ± 9.9	UC	Truelove and Witts
Koutroubakis IE et al., 2004 (51), Greece (CD)	P-selectin	ng/ml	80	NR	39 ± NR	50	NR	39 ± NR	CD	CDAI
Koutroubakis IE et al., 2004 (51), Greece (UC)	P-selectin	ng/ml	80	NR	39 ± NR	54	NR	43 ± NR	UC	SCCAI
Andoh A et al., 2005 (52), Japan (CD)	P-selectin	ng/ml	25	12/13	29.2 ± 6.5	43	20/23	28.8 ± 8.5	CD	CDAI
Andoh A et al., 2005 (52), Japan (UC)	P-selectin	ng/ml	25	12/13	29.2 ± 6.5	44	23/21	30.3 ± 11.5	UC	CAI
Pamuk GE et al., 2006 (53), Turkey	E-selectin	ng/ml	19	7/12	39.3 ± 12	18	7/11	37.2 ± 15.7	UC	CAI
Ogawa N et al., 2008 (54), Japan	ICAM-1	ng/ml	39	19/20	41.3 ± 10.7	53	24/29	37.6 ± 12.8	UC	NR
Song WB et al., 2009 (55), China (CD)	ICAM-1	ng/ml	30	NR	NR	20	NR	NR	CD	NR
Song WB et al., 2009 (55), China (UC)	ICAM-1	ng/ml	30	NR	NR	49	NR	NR	UC	NR
Polińska B et al., 2011 (56), Poland	P-selectin	ng/ml	32	11/21	NR	16	9/7	NR	UC	Truelove and Witts
Smids C et al., 2017 (57), Netherlands	VCAM-1	ng/ml	20	4/16	30.5 ± 6.2	66	21/45	30.1 ± 4.5	CD	HBI, SES-CD
Cibor D et al., 2020 (13) Poland (CD)	E-selectin P-selectin	ng/ml ng/ml	40	20/20	33.5 ± 4.6	66	33/33	31.3 ± 2.3	CD	CDAI
Cibor D et al., 2020 (13)Poland (UC)	E-selectin P-selectin	ng/ml ng/ml	40	20/20	33.5 ± 4.6	56	28/28	37.8 ± 5.5	UC	Mayo
Yarur AJ et al., 2017 (58), United States	ICAM-1 VCAM-1	ng/ml ng/ml	NR	NR	NR	94	59/35	38.3 ± 15.5	CD	Endoscopic and histological mucosal healing
Yarur AJ et al., 2019 (59), United States	ICAM-1 VCAM-1	ng/ml ng/ml	NR	NR	NR	47	24/23	39 ± 16	CD	Endoscopic – active if ulcerations>5mm
Sano Y et al., 2024 (60), Japan	P-selectin	ng/ml	NR	NR	NR	54	29/25	44.3 ± 21.3	UC	Endoscopic – Mayo score
Petrović S et al., 2024 (61), Serbia (CD)	P-selectin	ng/ml	NR	NR	NR	13	7/6	38.5 ± 14.6	CD	Endoscopic - SES-CD
Petrović S et al., 2024 (61), Serbia (UC)	P-selectin	ng/ml	NR	NR	NR	49	29/20	44.1 ± 12.3	UC	Endoscopic – Mayo score
Górecka A et al., 2024 (62), Poland (CD)	E-selectin P-selectin	ng/ml ng/ml	NR	NR	NR	16	9/7	32.1 ± 9.6	CD	CDAI
Górecka A et al., 2024 (62), Poland (UC)	E-selectin P-selectin	ng/ml ng/ml	NR	NR	NR	30	18/12	33.4 ± 12.8	UC	Endoscopic – Mayo score

CAI, Clinical Activity Index; CDAI, Crohn’s Disease Activity Index; HBI, Harvey-Bradshaw Index; SCCAI, Simple Clinical Colitis Activity Index; SES-CD, Simple Endoscopic Score for Crohn’s Disease; NR, Not Reported.

**Table 2:** The Newcastle-Ottawa Scale.

Study, year	Selection				Comparability		Exposure		Summary	
	Is the case definition adequate?	Representativeness of the cases	Selection of controls	Definition of controls	Study controls for age, gender	Study controls for any additional factor	Ascertainment of exposure	Same method of ascertainment for cases and controls	Points	Risk of bias
Dippold et al., 1993 (38)	–	–	–	✱	–	–	✱	✱	3	high
Vainer B. et al., 1994 (39)	✱	✱	✱	✱	✱	–	✱	✱	7	low
Nielsen O.H. et al., 1994 (40)	✱	✱	✱	✱	✱	–	✱	✱	7	low
Jones S.C. et al., 1995 (41)	✱	✱	✱	✱	–	–	✱	✱	6	moderate
Patel R.T. et al, 1995 (42)	✱	✱	✱	✱	–	–	✱	✱	6	moderate
Göke M. et al., 1997 (43)	✱	–	✱	✱	–	–	✱	✱	5	moderate
Bhatii M. et al., 1998 (44)	✱	✱	✱	✱	✱	–	✱	✱	7	low
Vainer B. et al., 1998 (45)	✱	✱	✱	✱	✱	–	✱	✱	7	low
Seidelin J. B. et al., 1998 (46)	✱	✱	–	✱	–	–	✱	✱	5	moderate
Fägerstam J.P. et al., 2000 (47)	✱	✱	✱	✱	✱	–	✱	✱	7	low
Goggins M.G. et al., 2001 (48)	✱	✱	✱	✱	–	–	✱	✱	6	moderate
Vainer B. et al., 2003 (29)	✱	–	–	✱	–	–	✱	✱	4	moderate
Magro F. et al., 2004 (50)	✱	✱	✱	✱	✱	–	✱	✱	7	low
Koutroubakis I. E. et al., 2004 (51)	✱	✱	✱	✱	–	–	✱	✱	6	moderate
Andoh A. et al., 2005 (52)	✱	✱	✱	✱	✱	✱	✱	✱	8	low
Pamuk G.E. et al., 2006 (53)	✱	✱	✱	✱	✱	✱	✱	✱	8	low
Ogawa N. et al., 2008 (54)	✱	–	–	✱	✱	–	✱	✱	5	moderate
Song WB. et al., 2009 (55)	✱	✱	–	✱	–	–	✱	✱	5	moderate
Polińska B. et al., 2011 (56)	✱	✱	✱	✱	–	–	✱	✱	6	moderate
Smids C. et al., 2017 (57)	✱	✱	✱	✱	✱	–	✱	✱	7	low
Cibor D. et al., 2020 (13)	✱	✱	✱	✱	✱	✱	✱	✱	8	low
Yarur AJ et al., 2017 (58)	✱	✱	✱	✱	✱	–	✱	✱	7	low
Yarur AJ et al., 2019 (59)	✱	✱	✱	✱	–	–	✱	✱	6	moderate
Sano Y. et al., 2024 (60)	✱	✱	✱	✱	–	–	✱	✱	6	moderate
Petrović S. et al., 2024 (61)	✱	✱	✱	✱	✱	–	✱	✱	7	low
Górecka A. et al., 2024 (62)	✱	✱	✱	✱	✱	✱	✱	✱	8	low

The Newcastle–Ottawa Scale. A star (*) was awarded for each item in which the study met the specified criteria.

### 3.2 sICAM-1

**Figure 2:**
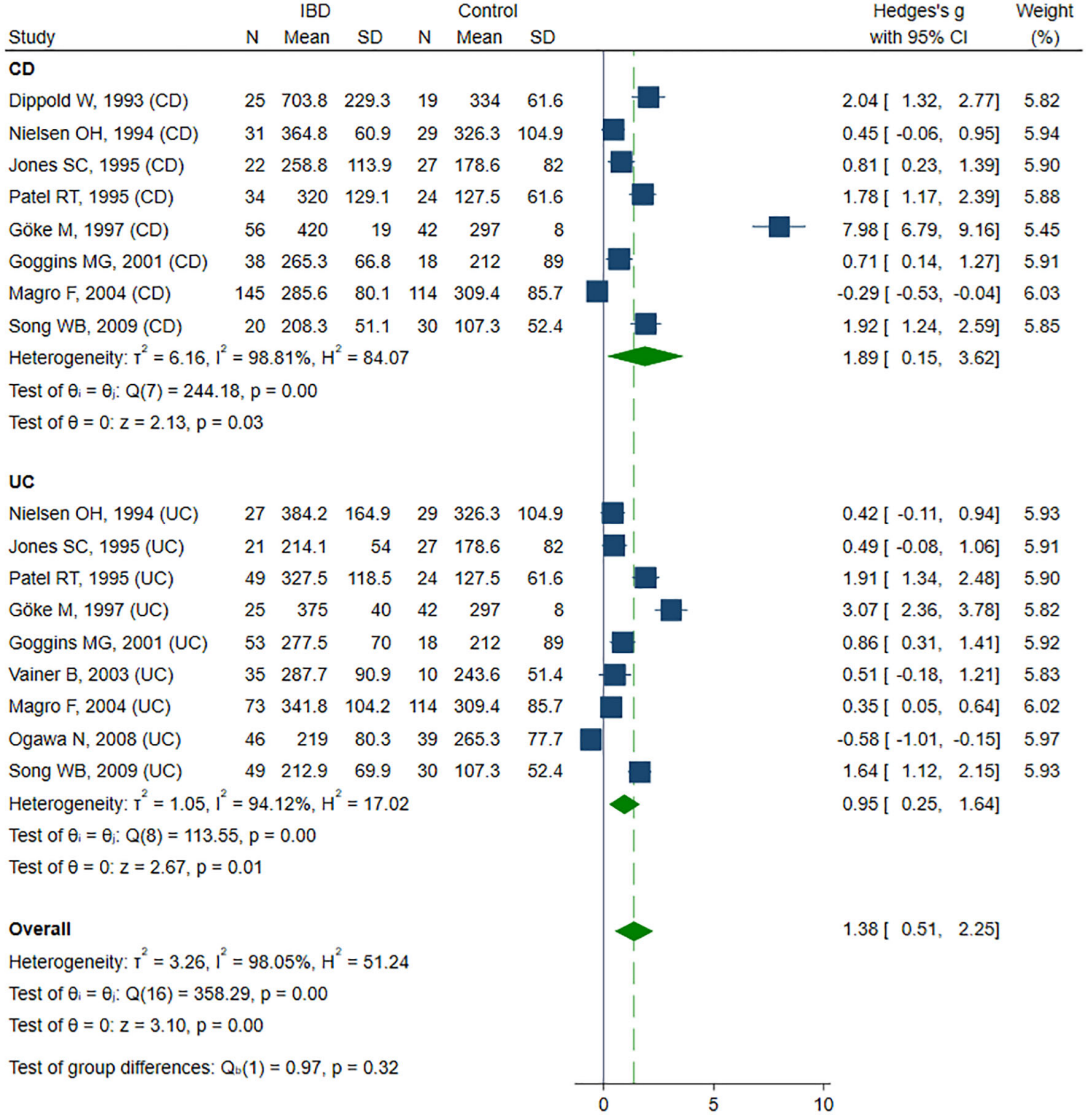
Forest plot of studies comparing sICAM-1 levels between IBD patients and healthy controls, with subgroup analysis for IBD.

Twelve studies (38, 40–43, 48–50, 54, 55, 58, 59) reported sICAM-1 concentrations, including a total of 364 healthy controls, 521 CD patients, and 388 UC patients. Seven studies (40–43, 48, 50, 55) included all three groups: healthy controls, CD, and UC patients; among these, five studies (40–42, 48, 50) further stratified the IBD groups according to disease activity. Two studies (39, 54) included only healthy controls and patients with inactive and active UC. One study (38) included healthy controls and patients with inactive and active CD. The remaining two studies (58, 59) compared only patients with inactive and active CD. The risk of bias was assessed as low in two studies (40, 50), moderate in nine studies (41–43, 48, 49, 54, 55, 58, 59), and high in one study (38) (**Table 2**). sICAM-1 concentration was significantly higher in the IBD group compared to controls (SMD 1.38, 95% CI 0.51 to 2.25, p=0.002; I² = 98.1%, p<0.001), and this association remained stable in the sensitivity analysis (SMD range 0.98 - 1.50). A significant publication bias was detected (Begg’s test, p<0.001; Egger’s test, p<0.001); however, the trim-and-fill method did not identify any potentially missing studies. When forced imputation was applied, six studies were imputed on the right side of the funnel plot, further increasing the overall SMD to 2.08 (95% CI 1.26 to 2.89). In the subgroup analysis, elevated sICAM-1 levels were consistently observed in both CD (SMD 1.89, 95% CI 0.15 to 3.62, p=0.033; I² = 98.8%, p<0.001) and UC patients (SMD 0.95, 95% CI 0.25 to 1.64, p=0.008; I² = 94.1%, p<0.001) (**Figure 2**). No difference in sICAM-1 concentrations was observed between the CD and UC groups (SMD -0.13, 95% CI -0.67 to 0.41, p=0.637; I² = 90.2%, p<0.001), and the result remained robust in the sensitivity analysis (SMD range -0.27 to 0.15). When comparing within the IBD group, sICAM-1 concentration was significantly higher in active IBD compared to inactive disease (SMD 0.75, 95% CI 0.38 to 1.12, p<0.001; I² = 81.4%, p<0.001), with no substantial changes observed in the sensitivity analysis (SMD range 0.59 - 0.82). Publication bias was not detected (Begg’s test, p=0.166; Egger’s test, p=0.088). Subgroup analysis demonstrated significantly higher sICAM-1 levels in active CD (SMD 0.92, 95% CI 0.39 to 1.45, p<0.001; I² = 83.2%, p<0.001) and UC (SMD 0.56, 95% CI 0.03 to 1.08, p = 0.037; I²=78.4%, p<0.001) compared to inactive disease.

### 3.3 sVCAM-1

**Figure 3:**
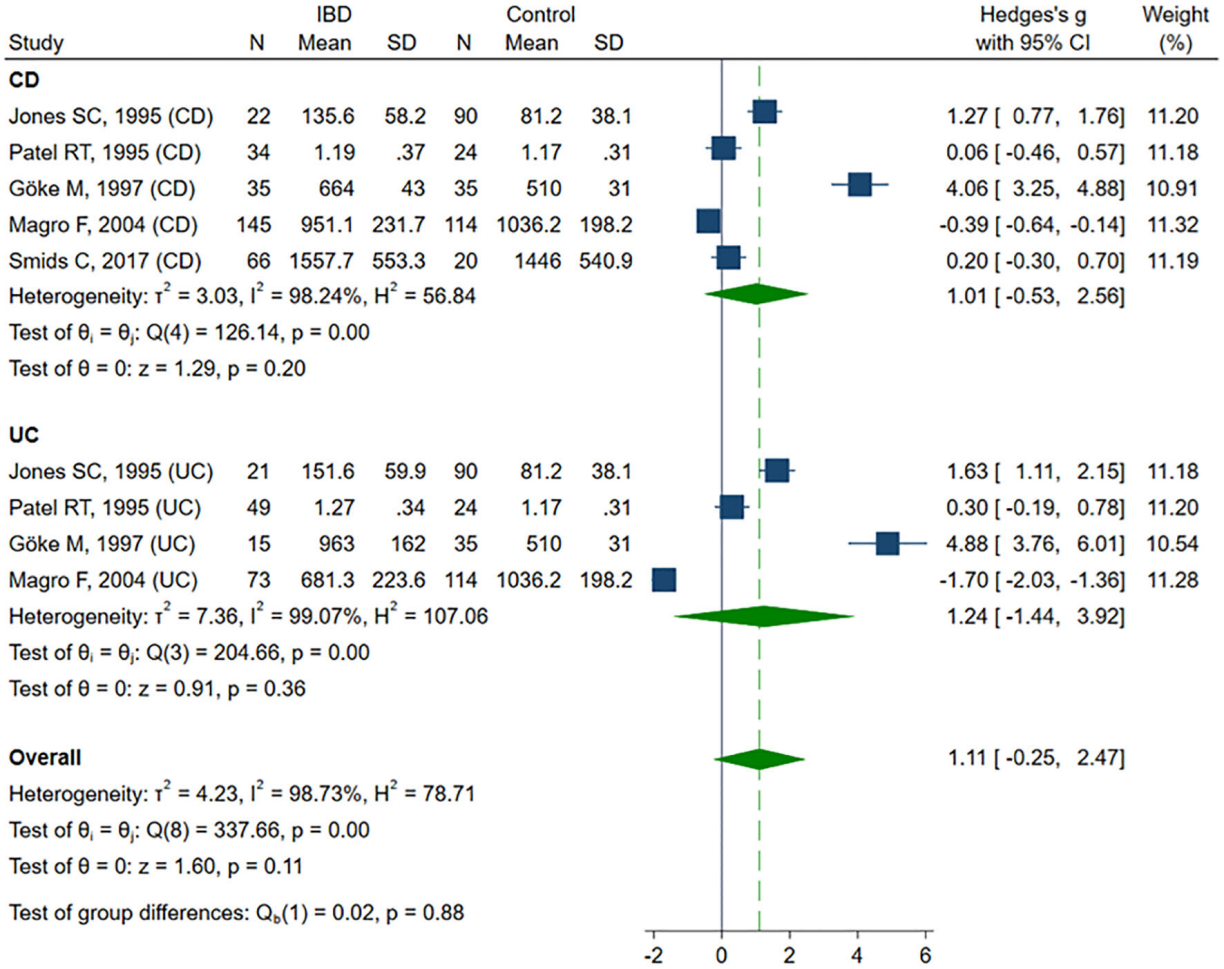
Forest plot of studies comparing sVCAM-1 levels between IBD patients and healthy controls, with subgroup analysis for IBD.

sVCAM-1 levels were evaluated across seven studies (41–43, 50, 57–59), including a total of 263 healthy controls, 443 CD patients and 158 UC patients. Four studies (41–43, 50) included healthy controls, CD and UC patients, and in three of them (41, 42, 50), IBD was subdivided according to disease activity. One study (57) included only active and inactive CD, along with controls. Two studies (58, 59) compared sVCAM-1 levels exclusively between active and inactive CD. The risk of bias was rated as low in three studies (50, 57, 58) and as moderate in four (41–43, 59) (**Table 2**). SMD indicated a trend toward elevated sVCAM-1 levels in IBD patients, although the result was not statistically significant (SMD 1.11, 95% CI –0.25 to 2.47, p=0.109; I² = 98.7%, p<0.001). In sensitivity analyses (SMD range from 0.74 to 1.46), exclusion of the UC subgroup from the Magro study (50) revealed a significant increase in sVCAM-1 levels among IBD patients (SMD 1.46, 95% CI 0.13–2.78, p=0.031). Removal of the entire Magro study further confirmed a significant elevation in sVCAM-1 levels (SMD 1.73, 95% CI 0.32–3.14; p=0.016; I² = 97.7%, p<0.001). Subgroup analysis, despite a consistent trend toward higher sVCAM-1 levels in patients with IBD, did not reveal statistically significant differences in SMD for CD (SMD 1.01, 95% CI –0.53 to 2.56, p=0.198; I² = 98.2%, p<0.001) and UC (SMD 1.24, 95% CI –1.44 to 3.92, p=0.363; I² = 99.1%; p< 0.001) compared with controls (**Figure 3**). Exclusion of the Magro study did not alter the statistical significance of these findings. No significant difference in sVCAM-1 levels was observed between CD and UC patients (SMD 0.58, 95% CI –1.16 to 2.31, p=0.516; I² = 98%, p<0.001), and the result remained stable in sensitivity analysis (SMD range from –0.25 to 1.17). Comparison between patients with active and inactive IBD also revealed no statistically significant difference in sVCAM-1 concentrations (SMD 0.20, 95% CI –0.11 to 0.51, p=0.212; I² = 63.5%, p = 0.003). This finding was consistent in sensitivity analysis (SMD ranged from 0.09 to 0.29), although exclusion of the UC subgroup from the Patel study (42) resulted in a borderline significant difference favoring higher sVCAM-1 levels in the active disease group (SMD 0.29, 95% CI –0.001 to 0.58, p=0.050). Subgroup analyses showed no significant differences in sVCAM-1 levels between inactive and active disease in either CD (SMD 0.24, 95% CI –0.08 to 0.56, p=0.140; I² = 50.0%, p=0.071) or UC (SMD 0.24, 95% CI –0.75 to 1.22, p=0.636; I² = 86.8%, p=0.004).

### 3.4 sMAdCAM-1

No studies comparing sMAdCAM-1 concentrations between the investigated groups were identified.

### 3.5 sE-selectin

**Figure 4:**
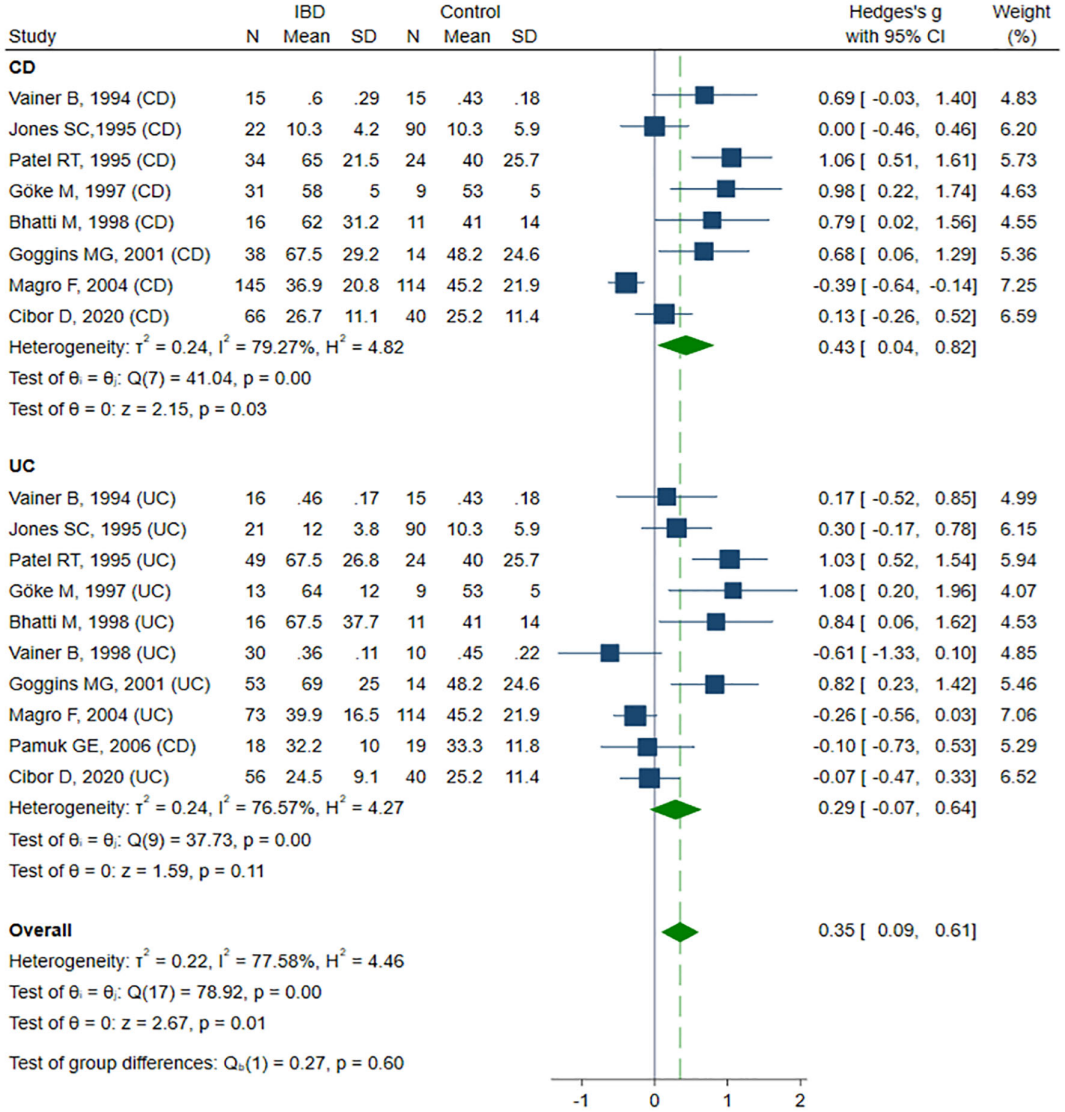
Forest plot of studies comparing sE-selectin levels between IBD patients and healthy controls, with subgroup analysis for IBD.

Eleven studies reported sE-selectin concentrations (13, 39, 41–45, 48, 50, 53, 62) in a total of 346 healthy controls, 383 CD patients, and 375 UC patients. Eight studies (13, 39, 41–44, 48, 50) included healthy controls, CD, and UC patients, of which six (13, 41, 42, 44, 48, 50) compared groups by disease activity. Two studies (45, 53) compared healthy controls with patients with active and inactive UC. One study (62) included only patients with active CD and UC. The risk of bias was assessed as low in seven studies (13, 39, 44, 45, 50, 53, 62), and moderate in four studies (41–43, 48) (**Table 2**). sE-selectin levels were significantly higher in patients with IBD compared to controls (SMD 0.35, 95% CI 0.09 to 0.61, p=0.008; I² = 77.6%, p<0.001), and this difference remained stable in the sensitivity analysis (SMD range 0.30 – 0.40). Significant publication bias was detected (Begg’s test, p=0.049; Egger’s test, p=0.002). Using the trim-and-fill method, four potentially missing studies were imputed on the left side of the funnel plot; their inclusion reduced SMD and resulted in a loss of statistical significance (SMD 0.17, 95% CI -0.09 to 0.44, p=0.208). In the subgroup analysis, elevated sE-selectin levels remained significant in the CD group (SMD 0.43, 95% CI 0.04 to 0.82, p=0.032; I² = 79.3%, p<0.001), but lost statistical significance in the UC group (SMD 0.29, 95% CI -0.07 to 0.64, p=0.113; I² = 76.6%, p<0.001) (**Figure 4**). No significant differences in sE-selectin levels were observed between CD and UC groups (SMD -0.05, 95% CI -0.42 to 0.32, p=0.803; I² = 81.2%, p<0.001), and this finding remained robust in the sensitivity analysis (SMD range -0.13 to 0.09). The study by Górecka et al. (62) was identified as a significant outlier; its exclusion markedly reduced heterogeneity, but without affecting the statistical significance of the results (SMD 0.09, 95% CI -0.11 to 0.28, p=0.396; I² = 30.3%, p=0.107). No significant differences in sE-selectin concentrations were observed between patients with active and inactive IBD (SMD 0.44, 95% CI -0.15 to 1.03, p=0.146; I² = 91.7%, p<0.001). Exclusion of the Vainer (45) (SMD 0.55, 95% CI -0.03 to 1.13, p=0.061) or Pamuk (53) (SMD 0.55, 95% CI -0.01 to 1.12, p=0.056) study shifted the elevated sE-selectin levels in active IBD closer to statistical significance; however, the results remained non-significant in sensitive analysis (SMD range 0.26 – 0.55). No publication bias was detected (Begg’s test, p=0.661, Egger’s test, p=0.051). Subgroup analysis did not reveal statistical significance between patients with active and inactive forms of CD (SMD 0.80, 95% CI -0.05 to 0.1.64, p=0.065; I² = 91.2%, p<0.001) and UC (SMD 0.16, 95% CI -0.66 to 0.98, p=0.699; I² = 91.7%, p<0.001).

### 3.6 sP-selectin

**Figure 5:**
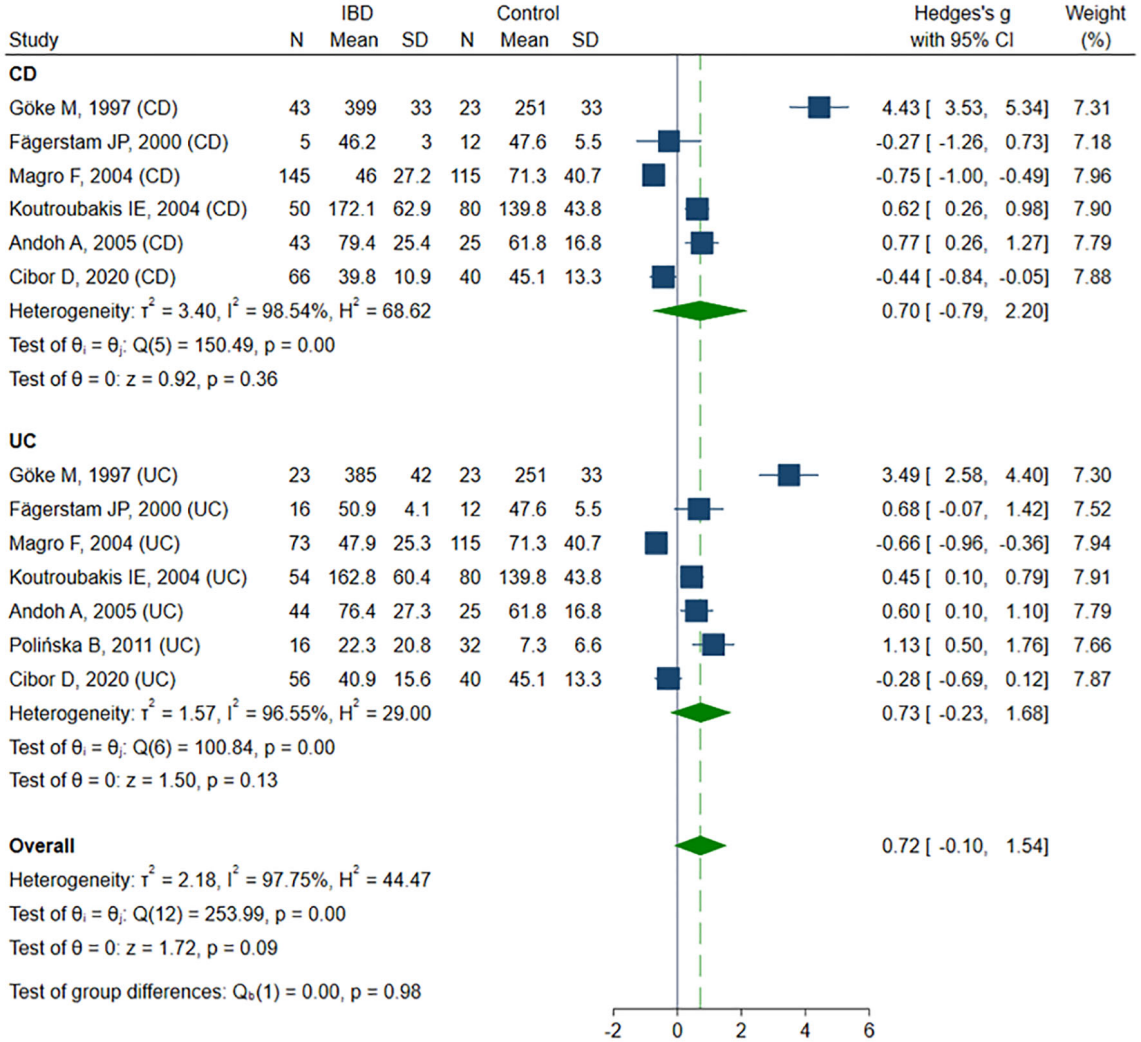
Forest plot of studies comparing sP-selectin levels between IBD patients and healthy controls, with subgroup analysis for IBD.

Ten studies (13, 43, 47, 50–52, 56, 60– 62) sP-selectin concentrations, including a total of 327 healthy controls, 309 CD patients, and 487 UC patients. Six studies (13, 43, 47, 50–52) included healthy controls, CD, and UC patients, among these, three studies (13, 50, 52) compared groups according to disease activity. One study (56) included controls and UC patients. The remaining three studies did not include healthy controls: in one study (61) sP-selectin levels were compared between active and inactive CD and UC patients, in one study (60), between active and inactive UC patients, and in one study (62) only in active CD and UC patients. The risk of bias was assessed as low in six studies (13, 47, 50, 52, 61, 62), and moderate in four studies (43, 51, 56, 60) (**Table 2**). Despite a tendency toward higher sP-selectin concentrations in IBD patients compared to the healthy controls, the difference was not statistically significant (SMD 0.72, 95% CI -0.10 to 1.54, p=0.086; I² = 97.8%, p<0.001). The results were stable in sensitivity analysis, with SMD ranging from 0.41 to 0.85. A significant publication bias was detected (Begg’s test, p=0.033; Egger’s test, p=0.002); however, the trim-and-fill method did not impute any missing studies. When forced imputation was applied, three studies were imputed on the right side of the funnel plot, resulting in a marked increase in the pooled SMD (95% CI 0.34 to 1.93, p=0.005). No differences in sP-selectin levels were observed in the CD (SMD 0.71, 95% CI -0.79 to 2.20, p=0.356; I² = 98.5%, p<0.001) and UC (SMD 0.73, 95% CI -0.23 to 1.68, p=0.135; I² = 96.6%, p<0.001) group in the subgroup analysis (**Figure 5**). No differences in sP-selectin concentrations were detected between patients with CD and UC (SMD -0.15, 95% CI -0.48 to 0.18, p=0.362; I² = 75.6%, p=0.002), with the finding remaining robust in the sensitivity analysis (SMD range -0.23 to -0.03). Similarly, no significant differences were found between active and inactive IBD patients (SMD 0.04, 95% CI -0.43 to 0.51, p=0.880; I² = 84.8%, p<0.001), and this result was maintained in subgroup analyses conducted for CD (SMD 0.27, 95% CI -0.08 to 0.67, p=0.129; I² = 40.5%, p=0.171), and UC (SMD 0.04, 95% CI -0.43 to 0.51, p=0.696; I² = 89.5%, p<0.001).

### 3.7 sL-selectin

Only one study (46) reported sL-selectin concentrations. sL-selectin levels did not differ significantly between healthy controls (n=12, median; IQR: 722; 634–767 ng/ml), patients with CD (n=16, 749; 613–846 ng/ml), and those with UC (n=23, 811; 611–874 ng/ml). However, within the UC subgroup, patients with inactive disease (n=8, 577; 428–632 ng/ml) demonstrated lower sL-selectin levels (p<0.005), while those with severe disease (n=7, 1012, 830–1068 ng/ml) exhibited elevated levels (p<0.002) compared to controls, with no differences with respect to mild and moderate activity subgroups. In contrast, no significant differences in sL-selectin levels were observed between CD patients - regardless of disease activity - and healthy individuals.

## 4 Discussion

The migration and adhesion of immune cells is a complex, multistep process involving a diverse set of molecules that are essential for effective immune and inflammatory responses. The initial phase of leukocyte adhesion involves transient and low-affinity interactions between selectins, L-selectin, P-selectin, and E-selectin, and their glycosylated ligands, mainly P-selectin glycoprotein ligand-1 (PSGL-1), which mediate leukocyte tethering and rolling along the endothelial surface under shear flow conditions. This rolling step is necessary for subsequent firm adhesion, primarily mediated by integrins such as α4β1 and α4β7. These integrins bind to immunoglobulin superfamily members expressed on activated endothelial cells, including ICAM-1, VCAM-1, and MAdCAM-1, allowing stable leukocyte arrest and transmigration (16, 63–66). Increased expression of these endothelial adhesion molecules has been consistently demonstrated in IBD patients, reflecting widespread endothelial activation within the inflamed intestinal mucosa (14, 20, 44, 67, 68).

Circulating forms of CAMs are produced through proteolytic shedding, and their elevated levels have been observed in various pathological conditions, including cardiovascular diseases, atherosclerosis, rheumatologic disorders, sepsis, and malignancies. These soluble isoforms likely reflect endothelial activation or injury and are increasingly investigated as potential biomarkers for disease activity, progression, and therapeutic response (17, 21–24, 69, 70).

Our study suggests a potential association between IBD, endothelial injury, and elevated levels of circulating adhesion molecules. Among the biomarkers analyzed, all showed a trend toward higher concentrations in IBD patients; however, only sICAM-1 and sE-selectin reached statistical significance. In subgroup analysis, the association for sICAM-1 remained significant in both CD and UC, while for sE-selectin, it persisted only in CD. This may be partially due to the fact that sICAM-1 and sE-selectin were evaluated in a greater number of studies compared to the other molecules. Importantly, only sICAM-1 was able to distinguish between active and inactive disease. None of the adhesion molecules showed significant differences between IBD subtypes.

We did not identify studies directly comparing sMAdCAM-1 levels between IBD patients and healthy controls. While no studies meeting the inclusion criteria specifically assessed vedolizumab or sMAdCAM-1, we briefly discuss available evidence from related literature as a narrative review. Notably, a few studies have explored the potential utility of sMAdCAM-1 as a biomarker for monitoring treatment response to vedolizumab. Vedolizumab is a humanized monoclonal IgG1 antibody that selectively inhibits the interaction between α4β7 integrin and MAdCAM-1, thereby blocking lymphocyte trafficking across the gut endothelium and reducing intestinal inflammation (20, 71, 72). Holmer et al. (73) demonstrated a reduction in sMAdCAM-1 levels in a cohort of 22 CD patients treated with vedolizumab; however, no significant differences were observed between patients who achieved remission and those who did not during the 26-week follow-up period. A similar association was reported by Battat et al. (74) in UC patients who presented with a decrease in sMAdCAM-1 levels compared to baseline, yet no significant differences were observed between remitters and non-remitters. However, Van den Berge et al. (75) and Kajikawa et al. (76) reported higher sMAdCAM-1 levels in patients with UC in remission at week 14 of treatment.

Vedolizumab, currently widely used in clinical practice, has demonstrated substantial efficacy and a favorable safety profile in the treatment of IBD by selectively targeting the α4β7 integrin-MAdCAM-1 pathway. Nevertheless, adhesion molecule pathways remain a focus of ongoing therapeutic development. Several emerging agents, such as natalizumab (targeting the α4 integrin subunit), etrolizumab (targeting the β7 integrin subunit), alicaforsen (inhibiting the activity of ICAM-1) and direct MAdCAM-1 inhibitors, are under investigation for their potential to further modulate leukocyte trafficking and improve clinical outcomes (77, 78). In this context, studies exploring the role of soluble adhesion molecules as biomarkers of endothelial activity and treatment response are of growing importance and may provide valuable tools for therapeutic monitoring and disease stratification.

### 4.1 Strengths and limitations

The main strength of our study was the evaluation of multiple adhesion molecules and the comparison of their concentrations not only between patients and healthy controls, but also across different types of IBD and disease activity states. Another strength was the use of subgroup analyses, as well as the application of the trim-and-fill method when appropriate and feasible, to identify potential missing studies.

Nevertheless, several important limitations should be acknowledged. First and foremost, only a small number of eligible studies were identified, most of which included relatively small sample sizes.

There was substantial heterogeneity among the included studies, which could not be fully explained. This variability may be attributed to several factors, including the overall moderate quality of the studies - as only half met the criteria for high quality according to the NOS scale — as well as the frequent lack of reporting of both basic demographic information and more detailed clinical characteristics, such as disease phenotype, duration, and treatment. Significant differences may also have arisen from the analytical methods employed, as various studies used ELISA kits from different manufacturers and analyzed different types of biological samples (serum vs. plasma), potentially contributing to discrepancies in the measured biomarker levels. Moreover, the high degree of heterogeneity may be partly related to the long time span of the included studies (1993–2025), during which laboratory techniques, diagnostic criteria, and the management of inflammatory bowel disease have evolved substantially.

Given the limited number of studies and incomplete reporting of individual patient data, it was not possible to perform a meta-regression analysis or adjust for potential confounding factors such as age, smoking status, specific medications, or disease duration. Furthermore, comparisons between IBD patients based on specific disease characteristics were not feasible, as participants were generally reported as a single group despite considerable variability in treatment regimens, disease phenotypes, and disease activity indices.

Significant publication bias was detected for sICAM-1 and sE-selectin. For sICAM-1, the bias did not affect the results and, in fact, the forced adjustment slightly increased the observed effect size. In contrast, for sE-selectin, the hypothetical missing studies suggested by the trim-and-fill method eliminated the statistical significance for the UC group.

Moreover, disease activity was assessed using different scoring systems, reflecting both the diversity of available tools and the evolution of assessment methods over the extended time span of the included studies. This variation in activity indices - including CDAI, Mayo, Truelove & Witts, and other scores - contributed substantially to clinical heterogeneity, as it made the classification of patients into “active” versus “inactive” disease groups less uniform across studies.

Finally, although a strength of our study was the inclusion of various adhesion molecules, it is important to acknowledge the multitude of other adhesion proteins that remain unexplored or are rarely measured, and therefore, could not be included in this meta-analysis.

## 5 Conclusions

Circulating CAMs, particularly sICAM-1 and sE-selectin, are elevated in IBD patients, supporting the involvement of endothelial injury in disease pathogenesis. Among these, sICAM-1 may have utility as a biomarker for differentiating disease activity. The complex mechanisms governing leukocyte adhesion and migration highlight opportunities for the development of targeted therapies that aim to modulate the inflammatory cascade. Circulating adhesion molecules may also serve as surrogate markers for evaluating therapeutic efficacy. However, given the limitations of the current evidence base and the multifactorial nature of these processes, further well-designed studies are needed to clarify their roles and enhance their translational potential in clinical practice.

## Data Availability

The original contributions presented in the study are included in the article/**Supplementary Material**. Further inquiries can be directed to the corresponding author/s.
