# Supplementary material for: Circulating cell adhesion molecules as biomarkers in inflammatory bowel disease: a systematic review and meta-analysis
Source: Front Immunol. 2025 Dec 1;16:1680317. doi: 10.3389/fimmu.2025.1680317 (PMC12702716; doi:10.3389/fimmu.2025.1680317)

frontiers

Supplementary Material

1. Search strategy

Database	Search query
PubMed	((ICAM[Title/Abstract]) OR (Intercellular Adhesion Molecule[Title/Abstract]) OR (sICAM[Title/Abstract]) OR (VCAM[Title/Abstract]) OR (sVCAM[Title/Abstract]) OR (Vascular cell adhesion molecule[Title/Abstract]) OR (Mucosal vascular addressin cell adhesion molecule[Title/Abstract]) OR (MAdCAM[Title/Abstract]) OR (sMAdCAM[Title/Abstract]) OR (selectin[Title/Abstract]) OR (L-selectin[Title/Abstract]) OR (sL-selectin[Title/Abstract]) OR (P-selectin[Title/Abstract]) OR (sP-selectin[Title/Abstract]) OR (E-selectin[Title/Abstract]) OR (sE-selectin[Title/Abstract]) OR (CD62L[Title/Abstract]) OR (CD62P[Title/Abstract]) OR (CD62E[Title/Abstract]) OR (sCD62L[Title/Abstract]) OR (sCD62P[Title/Abstract]) OR (sCD62E[Title/Abstract])) AND ((Crohn[Title/Abstract]) OR (ulcerative colitis[Title/Abstract]) OR (Inflammatory bowel disease[Title/Abstract]) OR (IBD[Title/Abstract]) OR (crohn's disease[Title/Abstract]))
Scopus	ABS(((ICAM) OR (Intercellular Adhesion Molecule) OR (sICAM) OR (VCAM) OR (sVCAM) OR (Vascular cell adhesion molecule) OR (Mucosal vascular addressin cell adhesion molecule) OR (MAdCAM) OR (sMAdCAM) OR (selectin) OR (L-selectin) OR (sL-selectin) OR (P-selectin) OR (sP-selectin) OR (E-selectin) OR (sE-selectin) OR (CD62L) OR (CD62P) OR (CD62E) OR (sCD62L) OR (sCD62P) OR (sCD62E)) AND ((Crohn) OR (ulcerative colitis) OR (Inflammatory bowel disease) OR (IBD) OR (crohn's disease)))
Embase	(icam:ab,ti OR 'intercellular adhesion molecule':ab,ti OR sicam:ab,ti OR vcam:ab,ti OR svcam:ab,ti OR 'vascular cell adhesion molecule':ab,ti OR 'mucosal vascular addressin cell adhesion molecule':ab,ti OR madcam:ab,ti OR smadcam:ab,ti OR selectin:ab,ti OR 'l selectin':ab,ti OR 'sl selectin':ab,ti OR 'p selectin':ab,ti OR 'sp selectin':ab,ti OR 'e selectin':ab,ti OR 'se selectin':ab,ti OR cd62l:ab,ti OR cd62p:ab,ti OR cd62e:ab,ti OR scd62l:ab,ti OR scd62p:ab,ti OR scd62e:ab,ti) AND (crohn:ab,ti OR 'ulcerative colitis':ab,ti OR 'inflammatory bowel disease':ab,ti OR ibd:ab,ti OR 'crohns disease':ab,ti)
Web of Science	AB=(((ICAM) OR (Intercellular Adhesion Molecule) OR (sICAM) OR (VCAM) OR (sVCAM) OR (Vascular cell adhesion molecule) OR (Mucosal vascular addressin cell adhesion molecule) OR (MAdCAM) OR (sMAdCAM) OR (selectin) OR (L-selectin) OR (sL-selectin) OR (P-selectin) OR (sP-selectin) OR (E-selectin) OR (sE-selectin) OR (CD62L) OR (CD62P) OR (CD62E) OR (sCD62L) OR (sCD62P) OR (sCD62E)) AND ((Crohn) OR (ulcerative colitis) OR (Inflammatory bowel disease) OR (IBD) OR (crohn's disease)))

2. Supplementary Figures

2.1 Supplementary Figures for sICAM-1.

2.1.1 Supplementary Figure 1. Forest plot of studies comparing sICAM-1 levels between IBD patients and healthy controls.

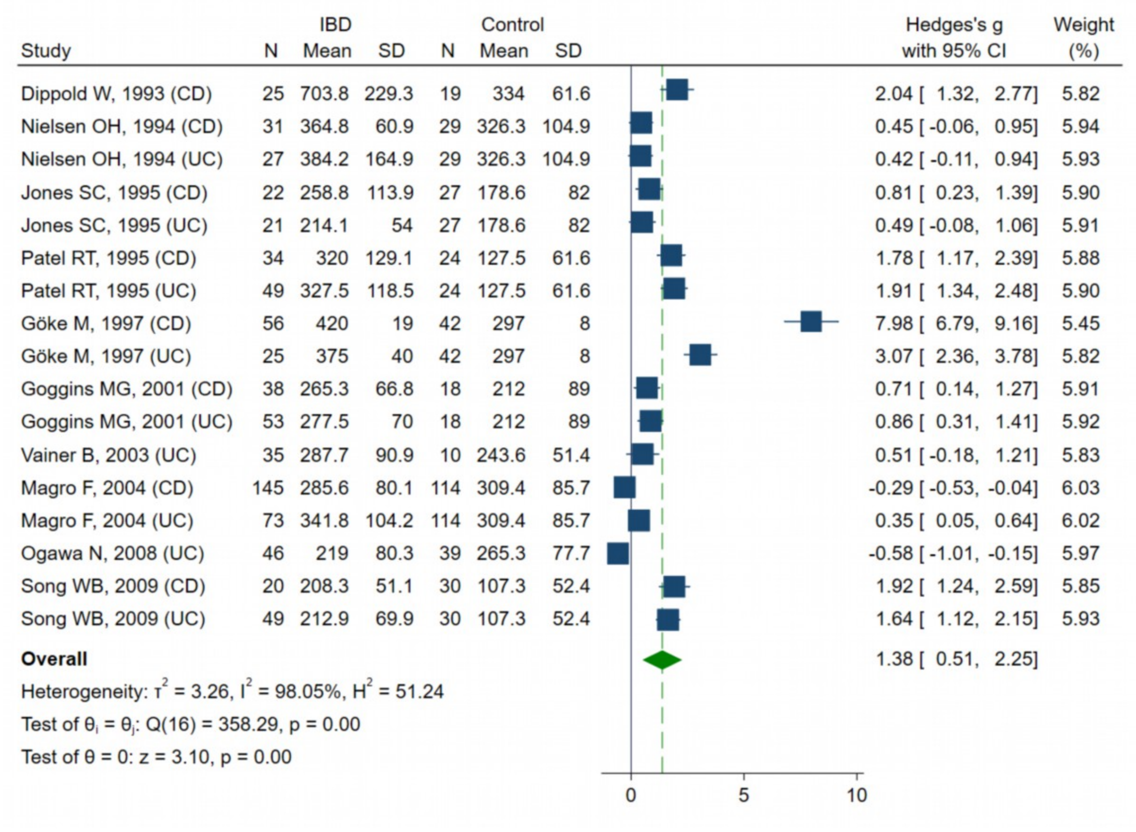

2.1.2 Supplementary Figure 2. Sensitive analysis of studies comparing sICAM-1 levels between IBD patients and healthy controls.

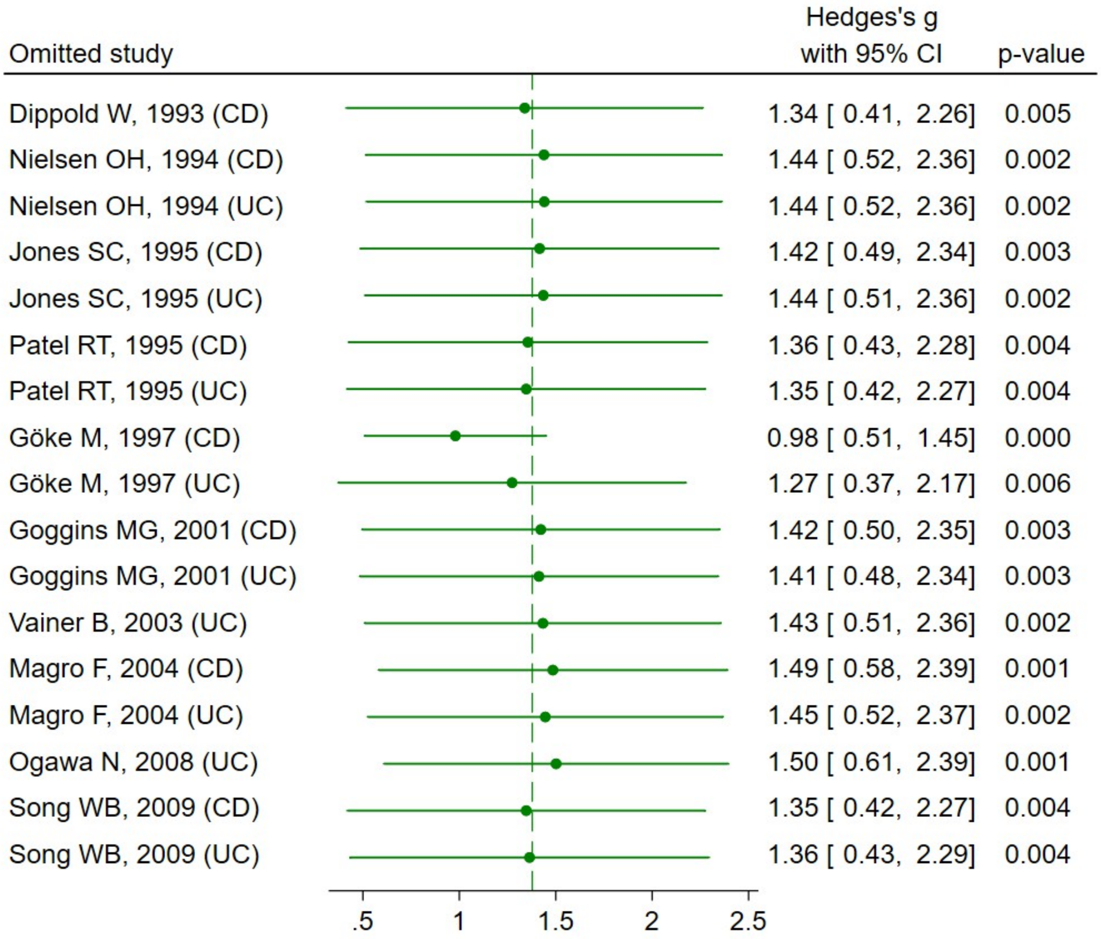

2.1.3 Supplementary Figure 3. Funnel plot of studies comparing sICAM-1 levels between IBD patients and healthy controls.

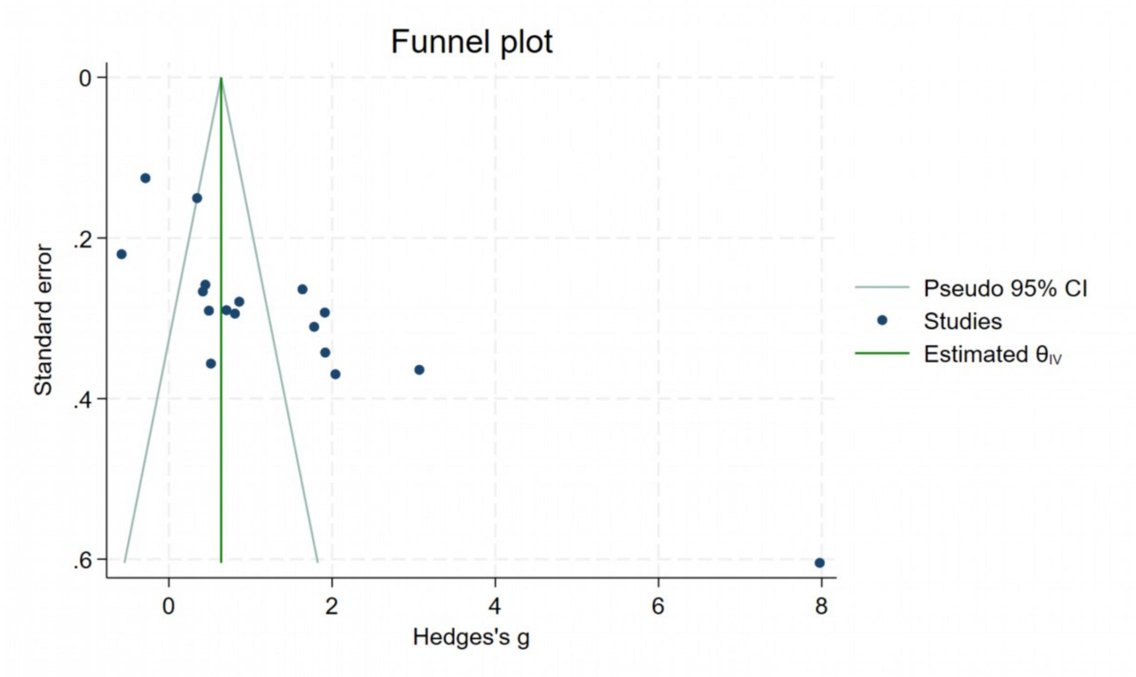

2.1.4 Supplementary Figure 4. Forest plot of studies comparing sICAM-1 levels between CD and UC patients.

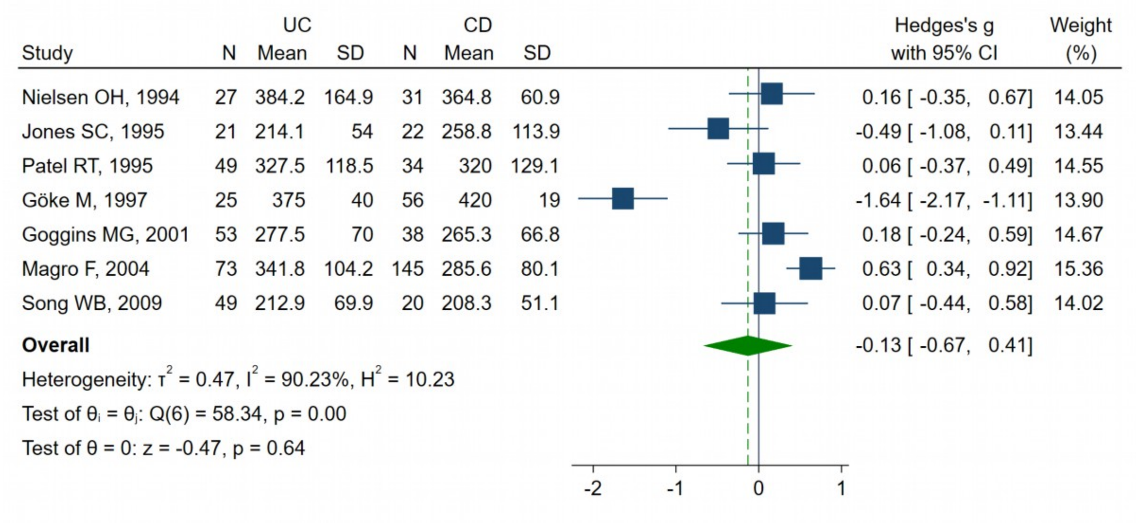

2.1.5 Supplementary Figure 5. Sensitive analysis of studies comparing sICAM-1 levels between CD and UC patients.

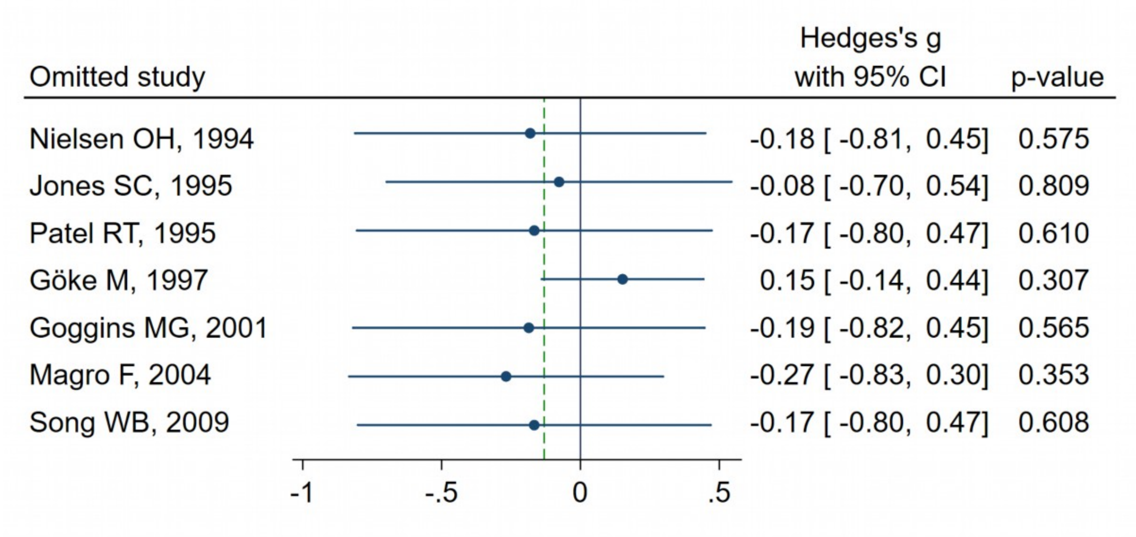

2.1.6 Supplementary Figure 6. Forest plot of studies comparing sICAM-1 levels between inactive and active IBD patients.

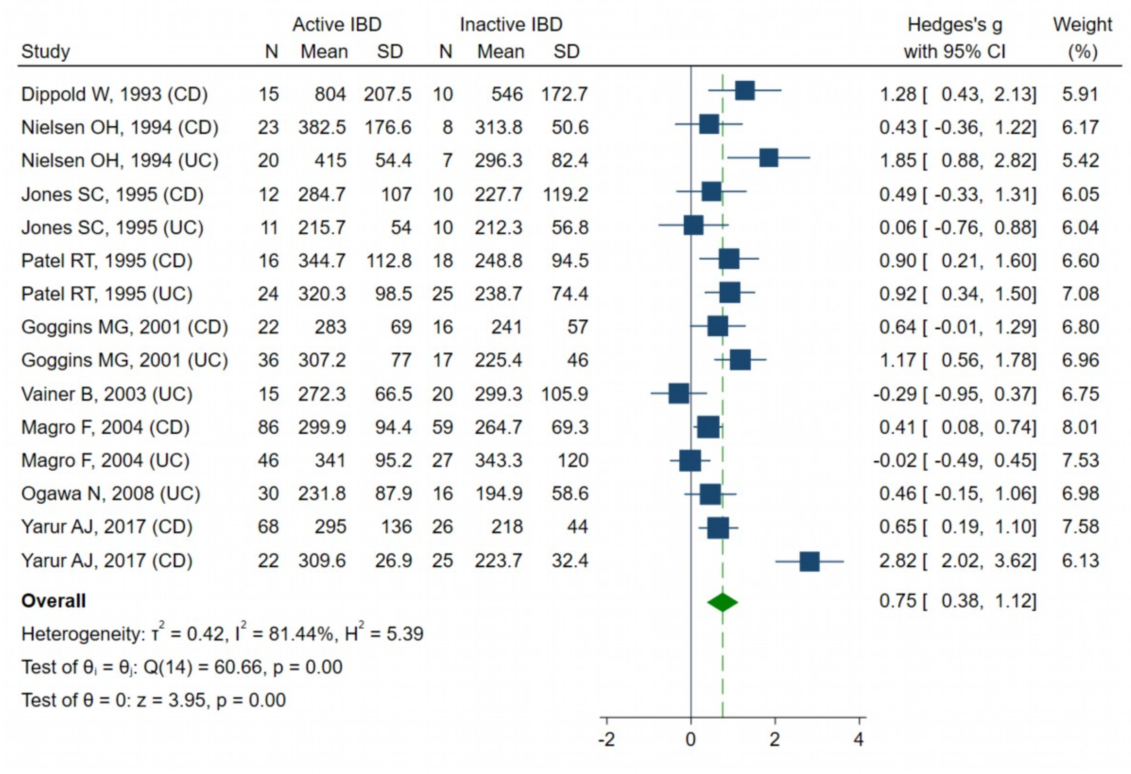

2.1.7 Supplementary Figure 7. Sensitive analysis of studies comparing sICAM-1 levels between inactive and active IBD patients.

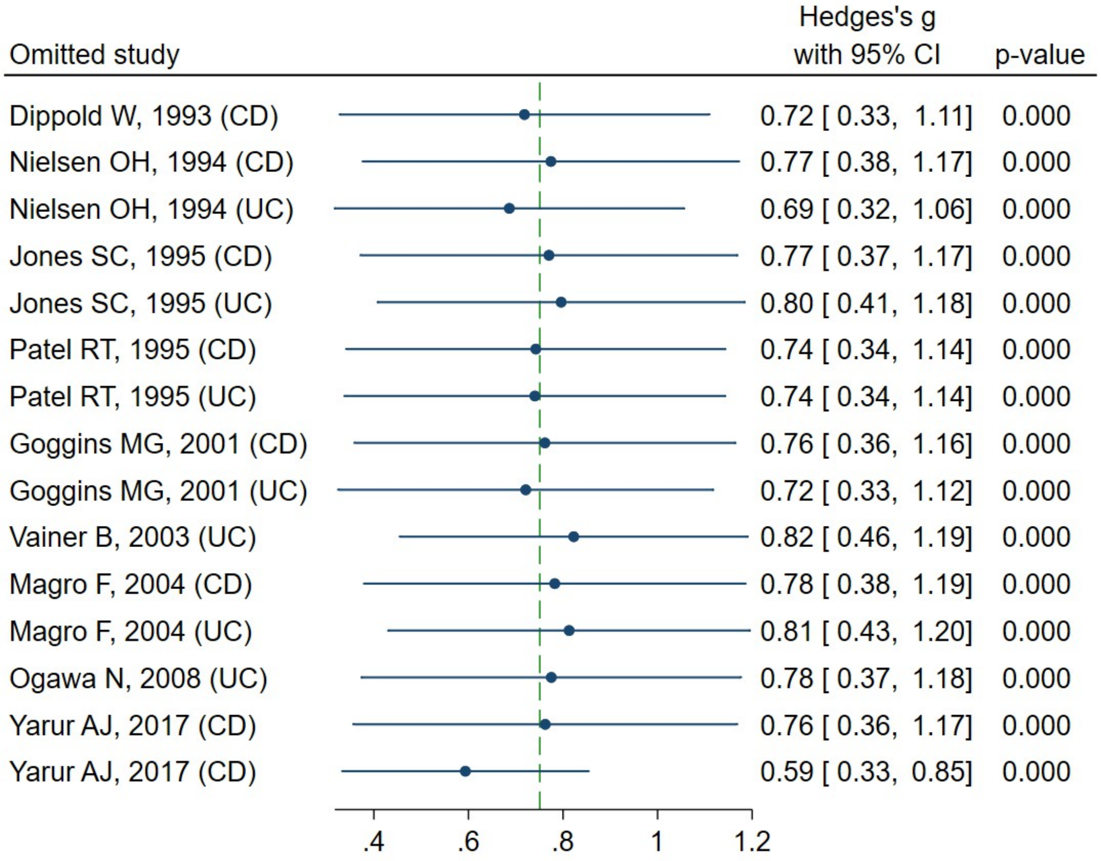

2.1.8 Supplementary Figure 8. Funnel plot of studies comparing sICAM-1 levels between inactive and active IBD patients.

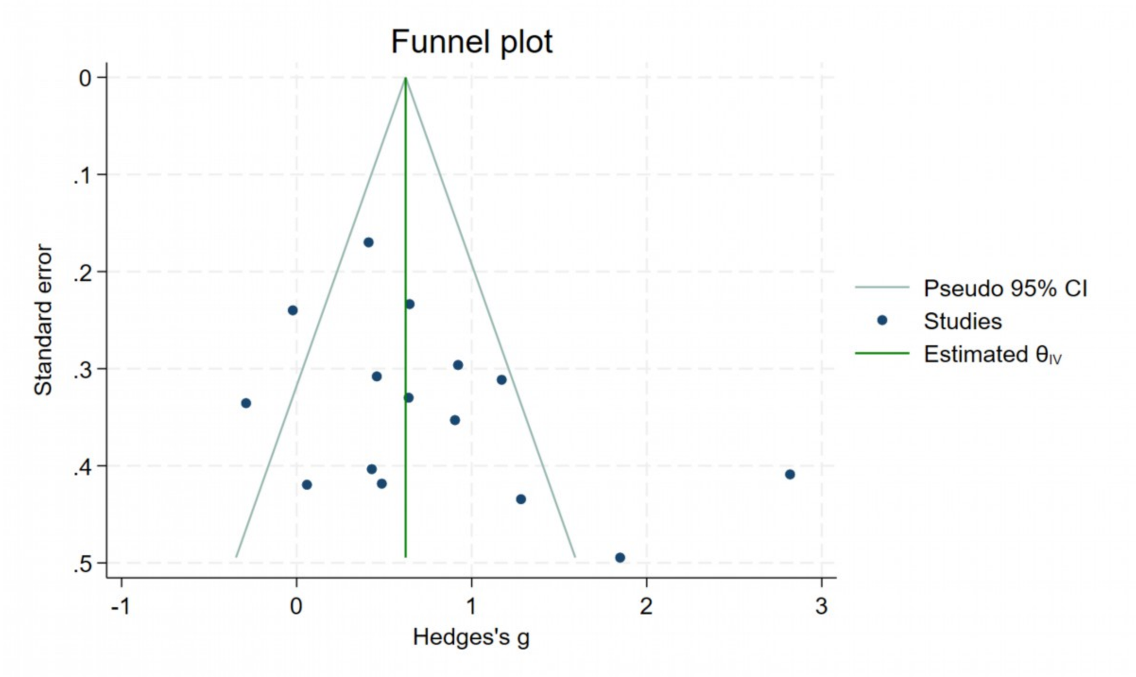

2.1.9 Supplementary Figure 9. Forest plot of studies comparing sICAM-1 levels between inactive and active IBD patients, with subgroup analysis for IBD.

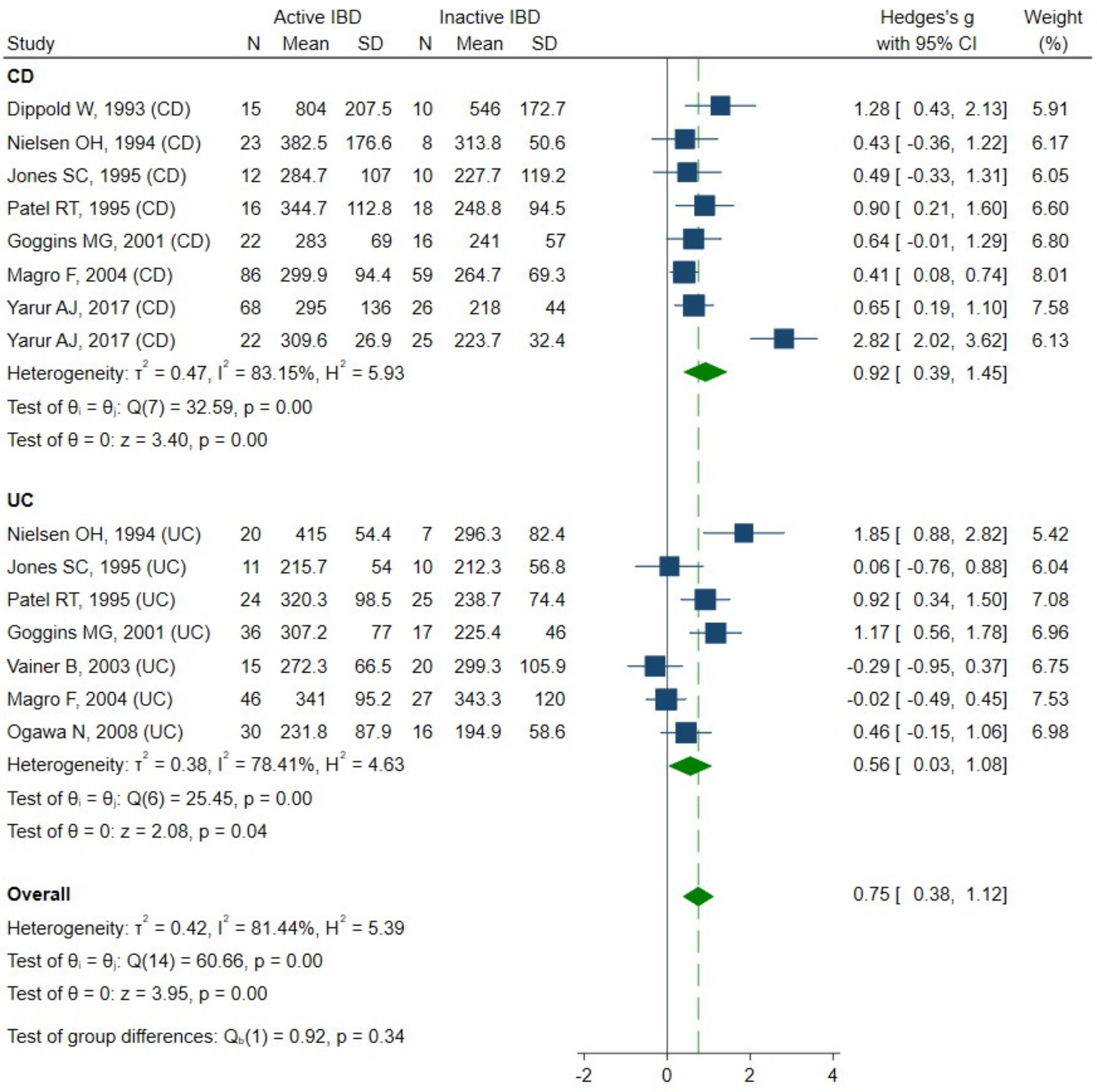

Supplementary Material

2.2 Supplementary Figures for sVCAM-1.

2.2.1 Supplementary Figure 10. Forest plot of studies comparing sICAM-1 levels between IBD patients and healthy controls.

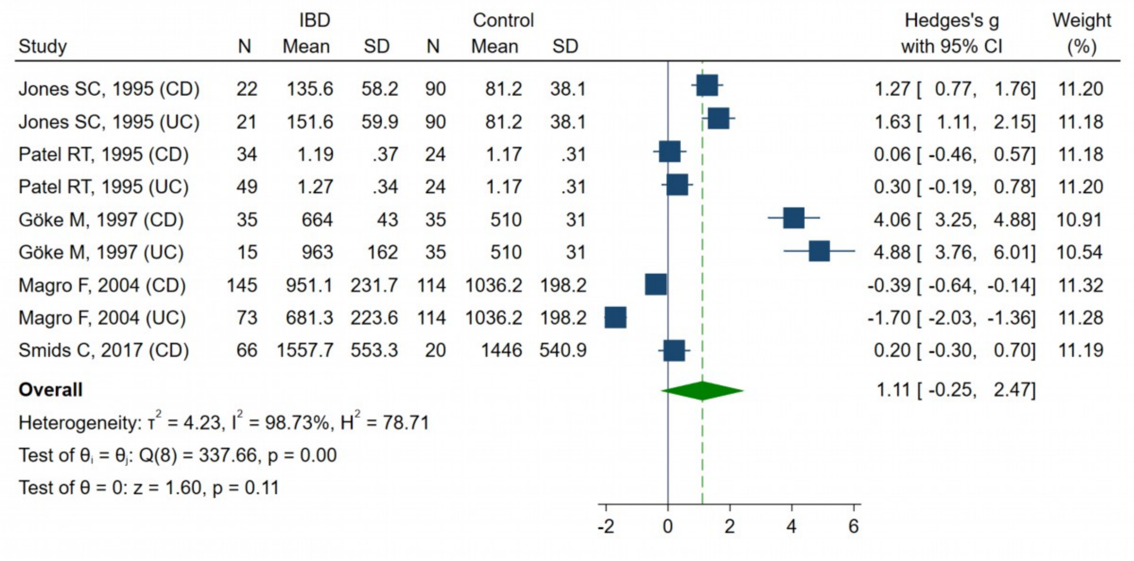

2.2.2 Supplementary Figure 11. Sensitive analysis of studies comparing sVCAM-1 levels between IBD patients and healthy controls.

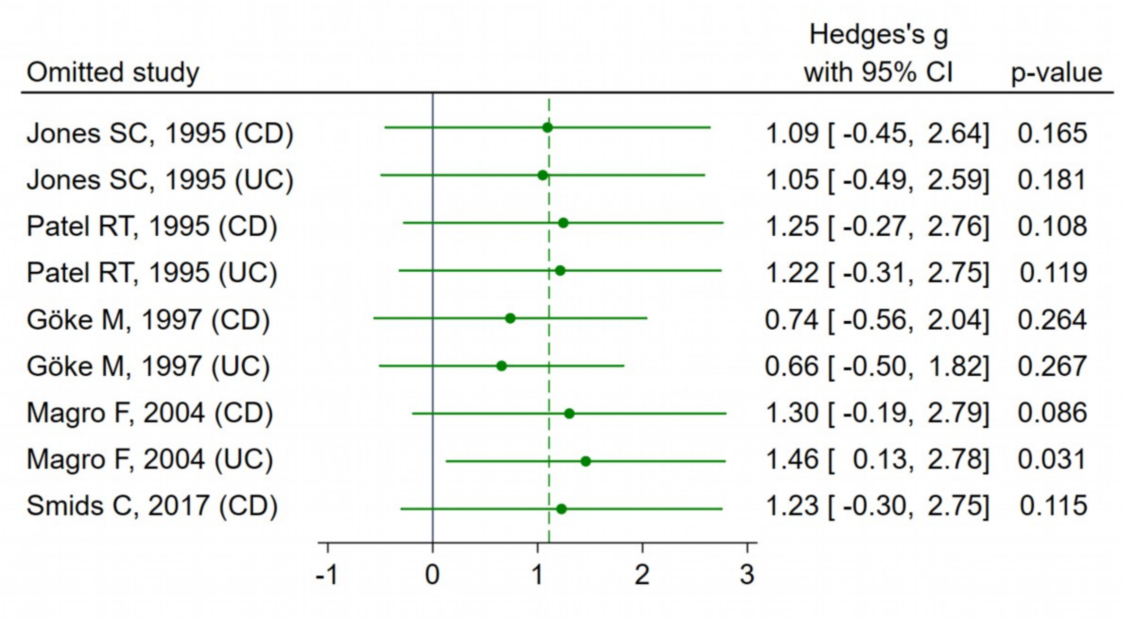

2.2.3 Supplementary Figure 12. Forest plot of studies comparing sVCAM-1 levels between CD and UC patients.

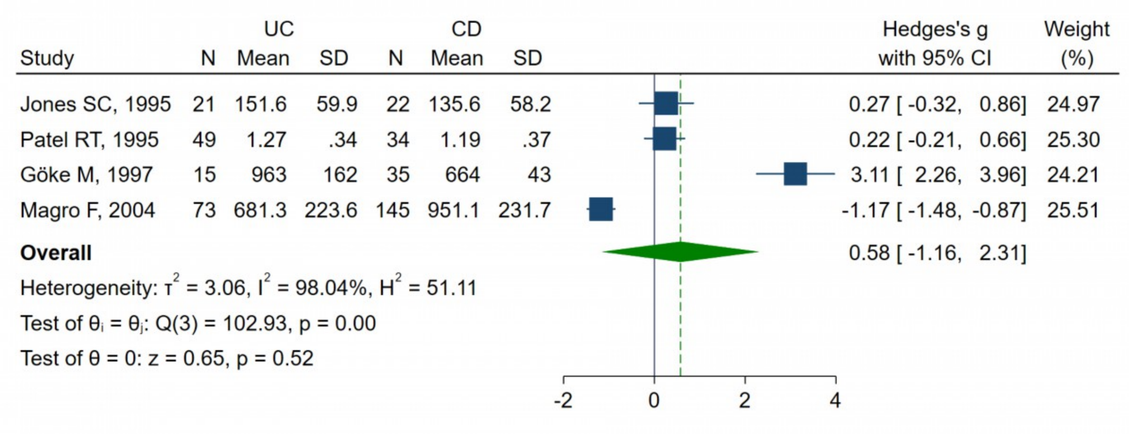

2.2.4 Supplementary Figure 13. Sensitive analysis of studies comparing sVCAM-1 levels between CD and UC patients.

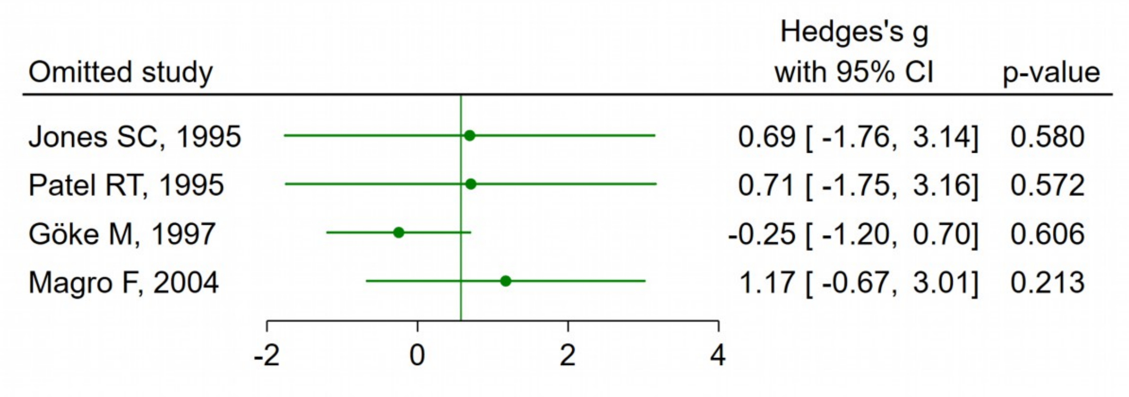

2.2.5 Supplementary Figure 14. Forest plot of studies comparing sVCAM-1 levels between inactive and active IBD patients.

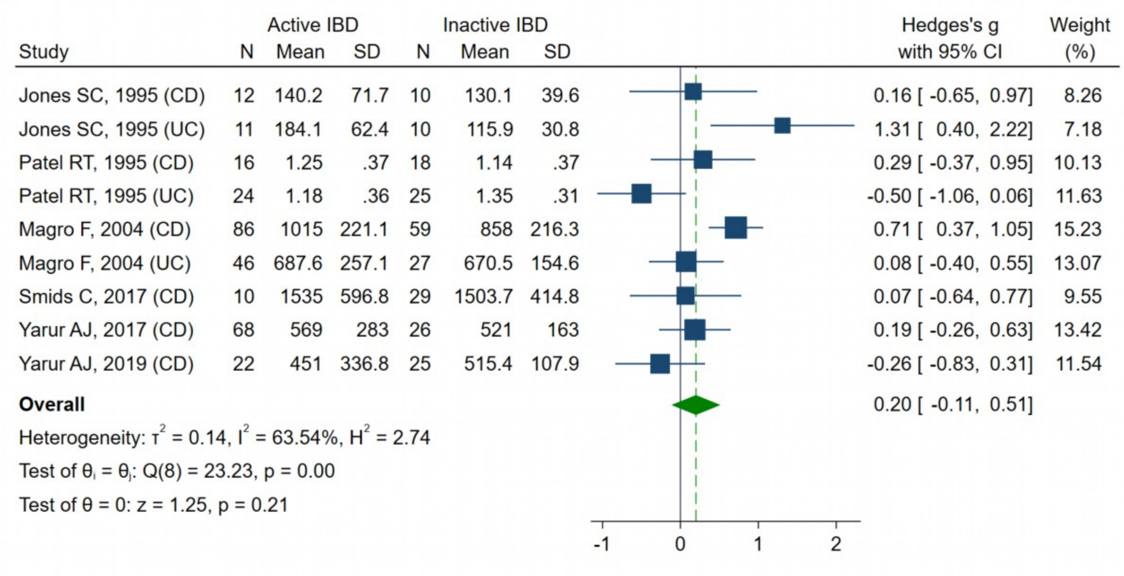

2.2.6 Supplementary Figure 15. Sensitive analysis of studies comparing sVCAM-1 levels between inactive and active IBD patients.

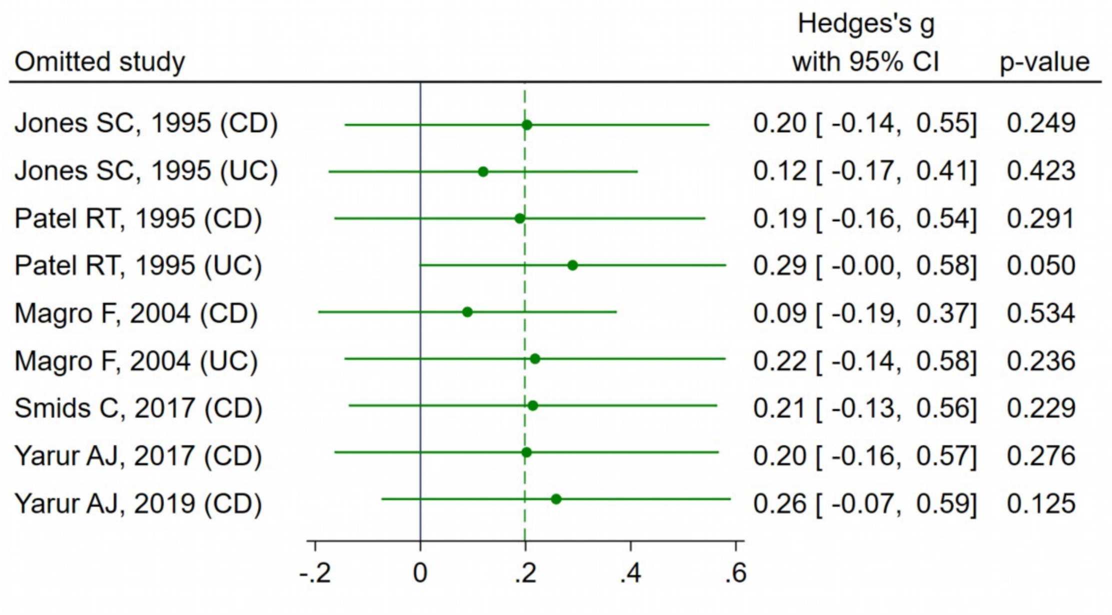

2.2.7 Supplementary Figure 16. Forest plot of studies comparing sVCAM-1 levels between inactive and active IBD patients, with subgroup analysis for IBD.

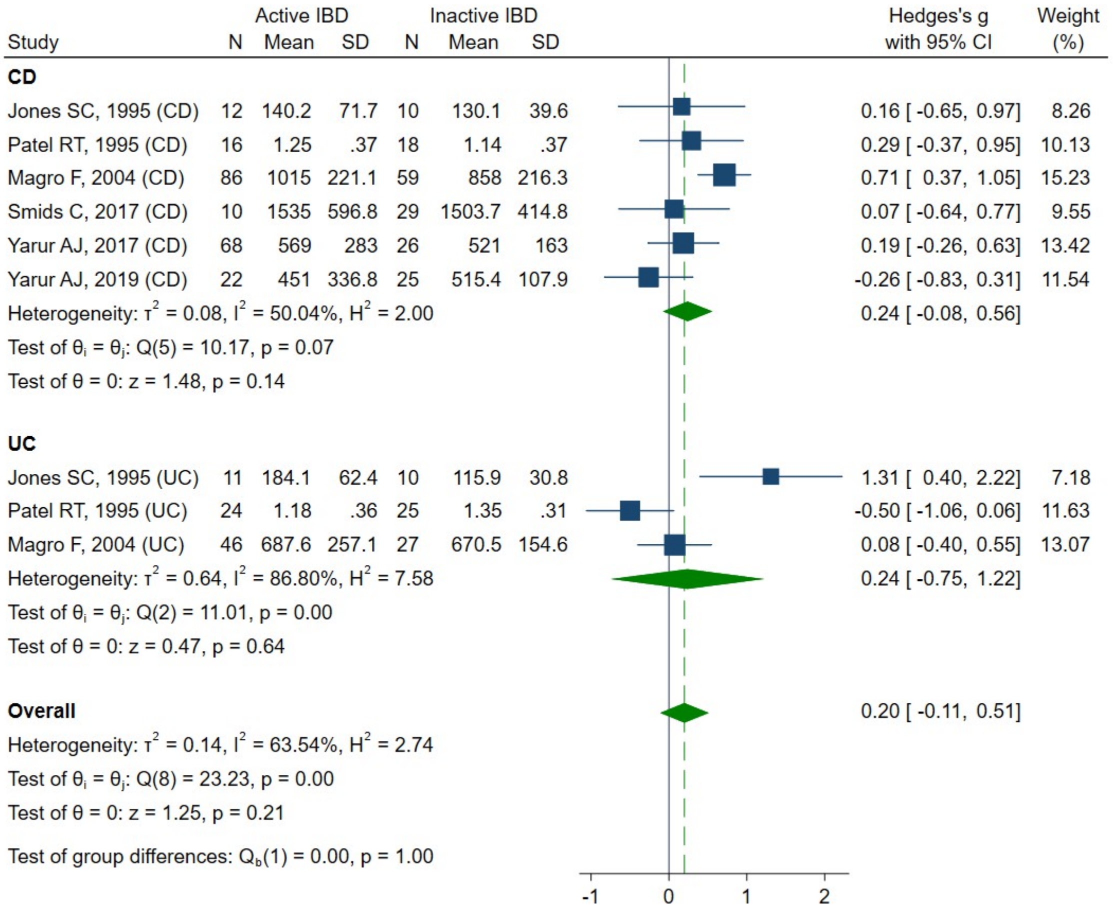

Supplementary Material

2.3 Supplementary Figures for sE-selectin.

2.3.1 Supplementary Figure 17. Forest plot of studies comparing sE-selectin levels between IBD patients and healthy controls.

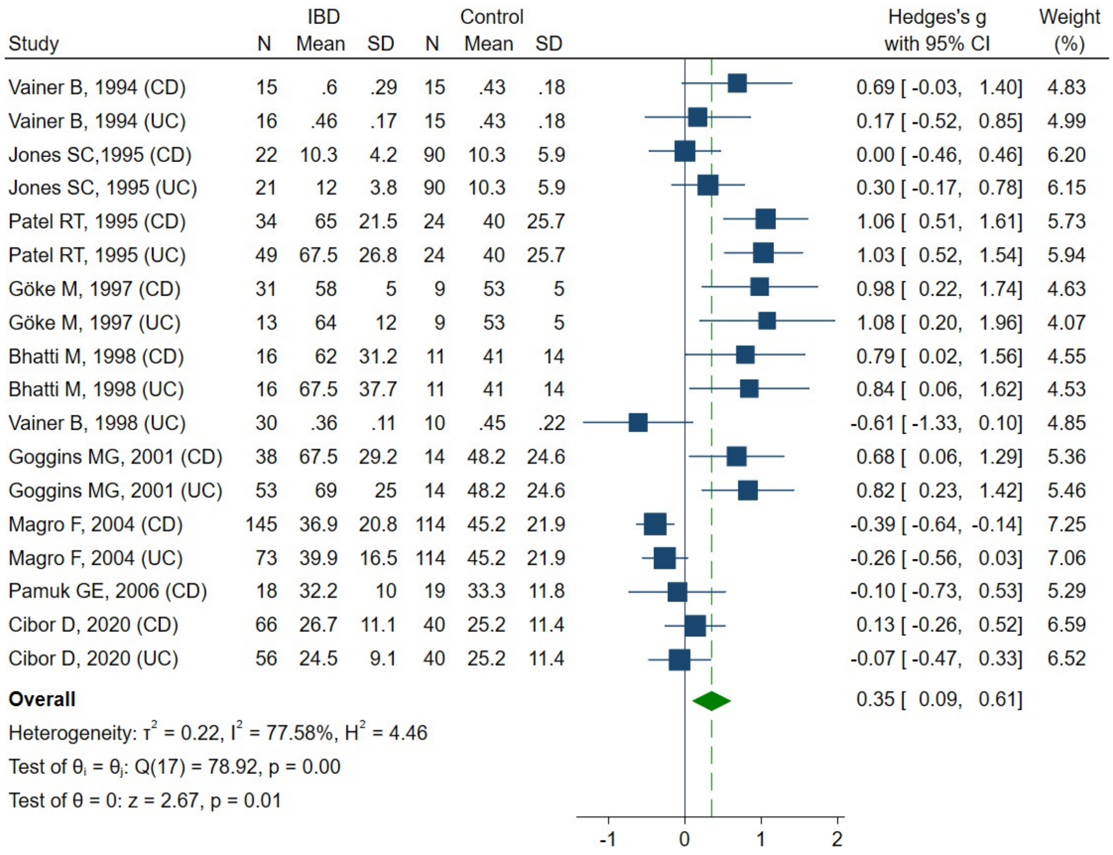

2.3.2 Supplementary Figure 18. Sensitive analysis of studies comparing sE-selectin levels between IBD patients and healthy controls.

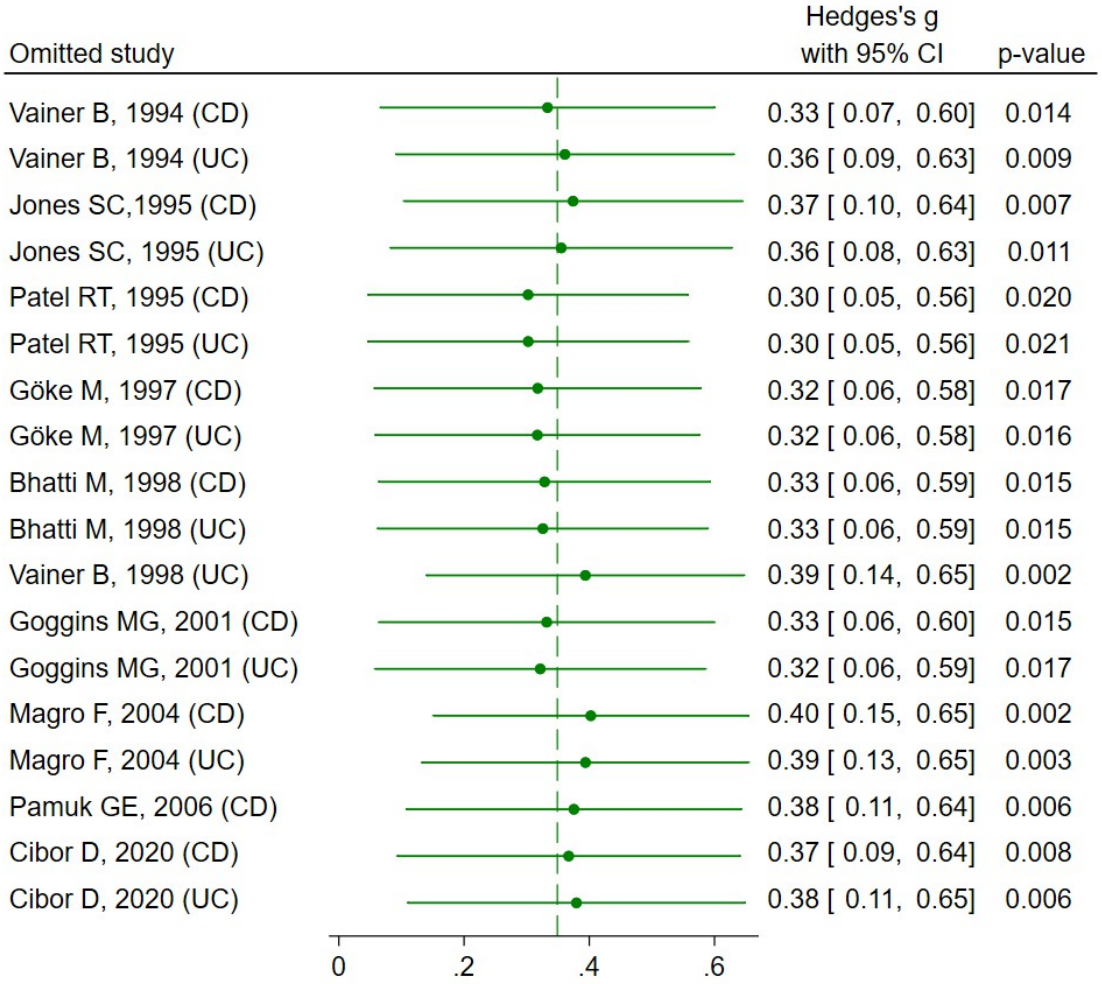

2.3.3 Supplementary Figure 19. Funnel plot of studies comparing sE-selectin levels between IBD patients and healthy controls.

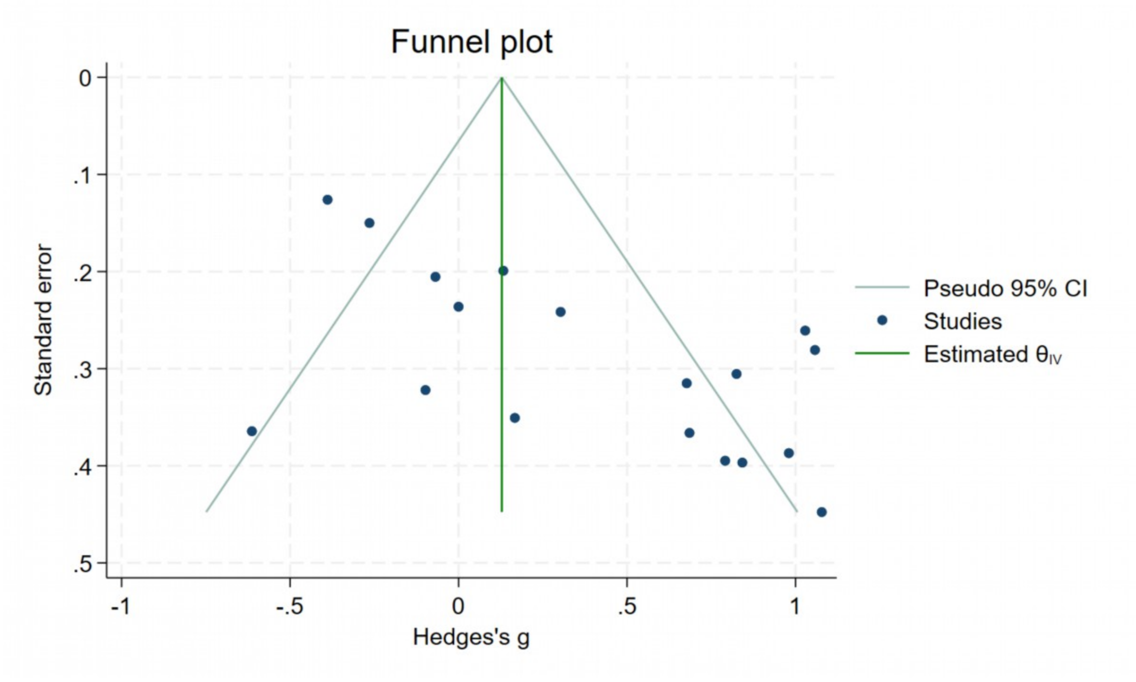

2.3.4 Supplementary Figure 20. Funnel plot, with trim and fill method, of studies comparing sE-selectin levels between IBD patients and healthy controls.

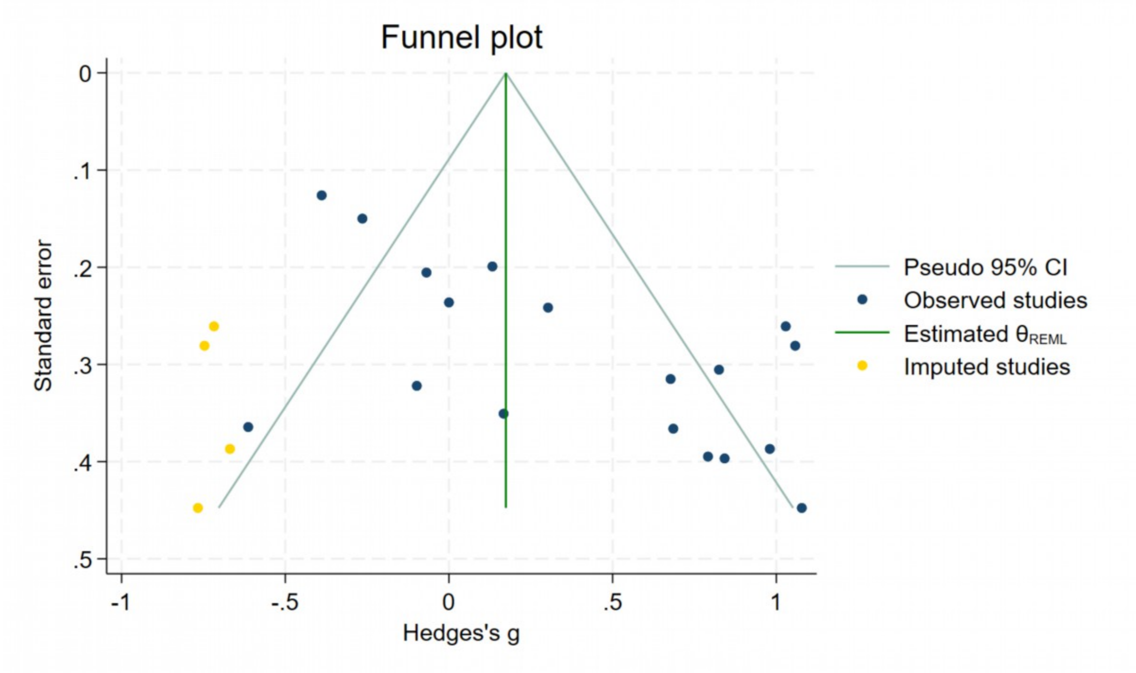

2.3.5 Supplementary Figure 21. Forest plot of studies comparing sE-selestin levels between CD and UC patients.

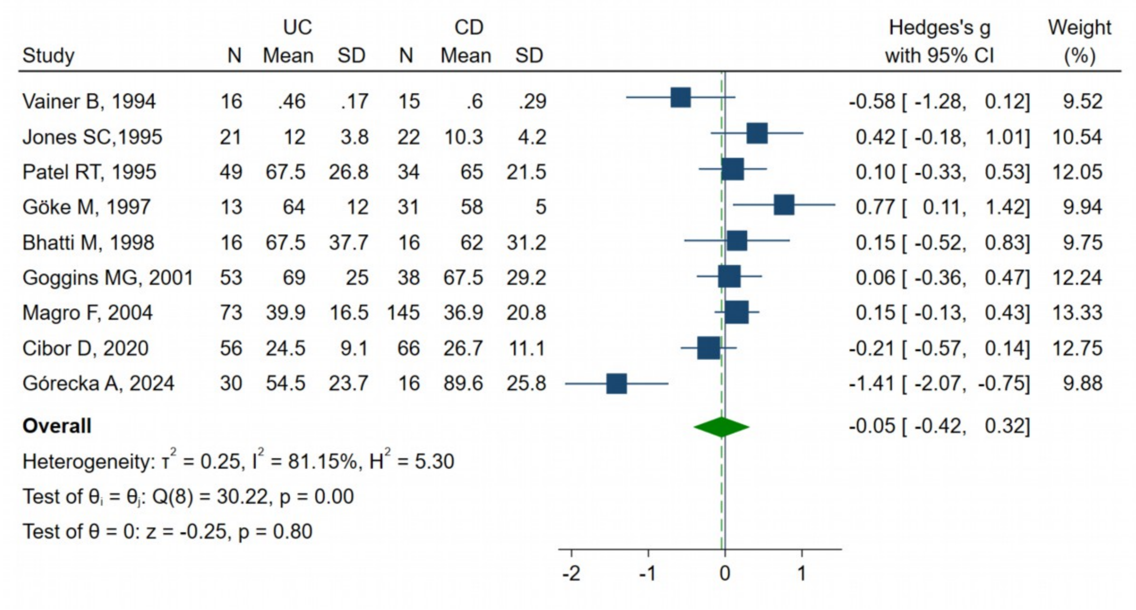

2.3.6 Supplementary Figure 22. Sensitive analysis of studies comparing sE-selectin levels between CD and UC patients.

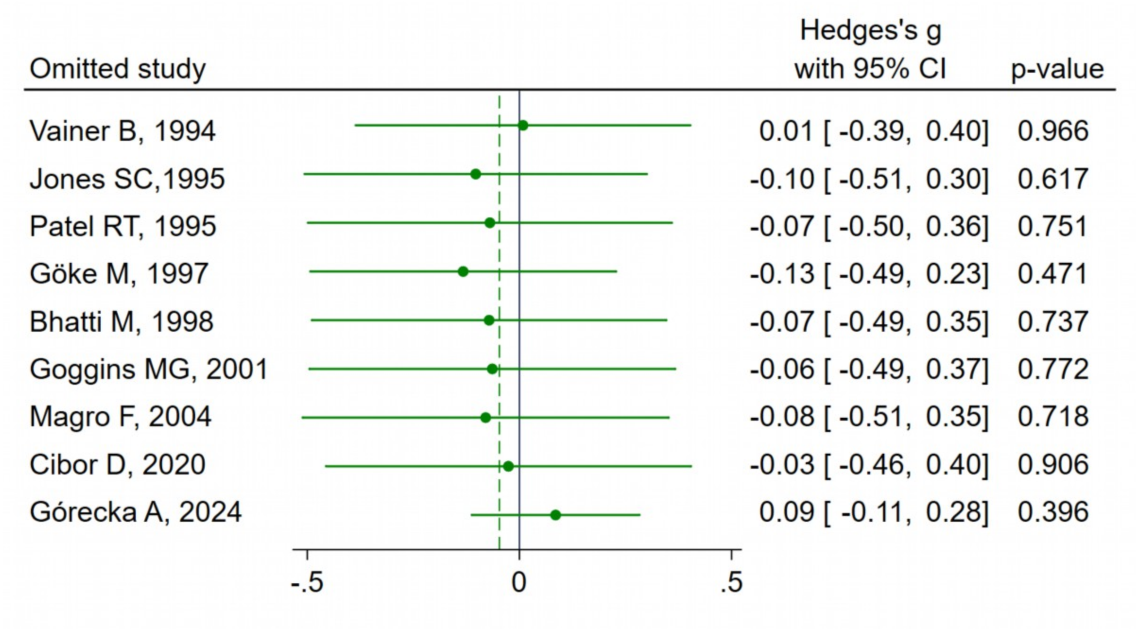

2.3.7 Supplementary Figure 23. Forest plot of studies comparing sE-selectin levels between inactive and active IBD patients.

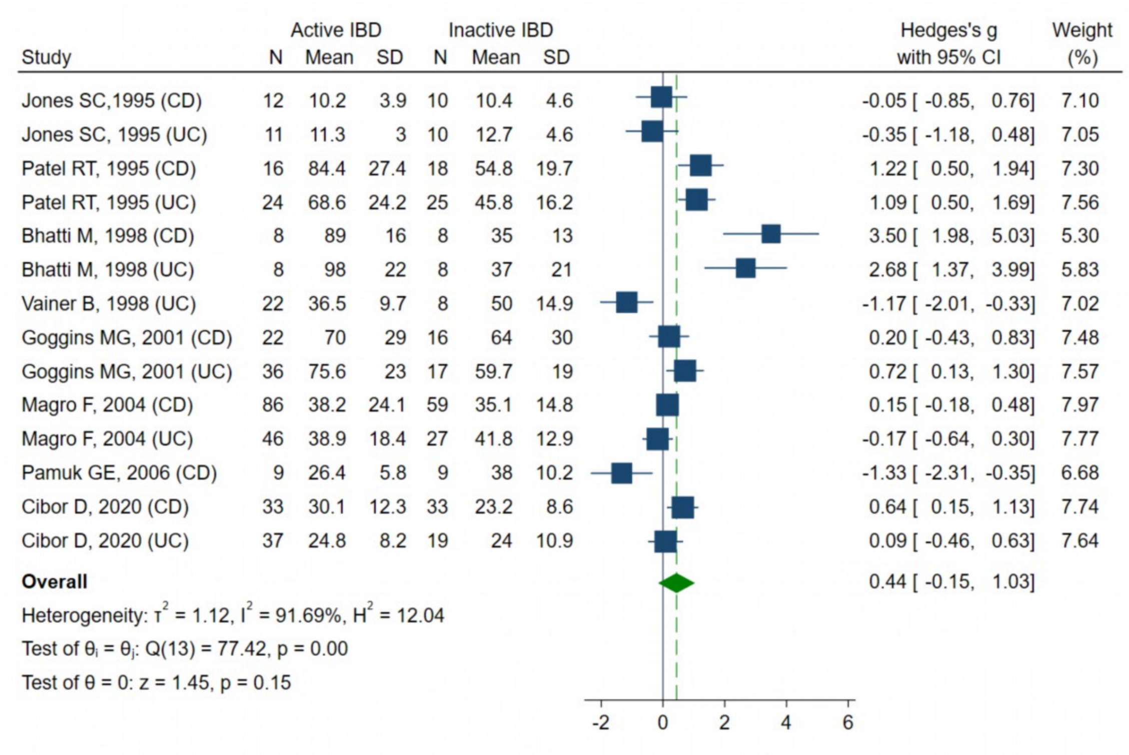

2.3.8 Supplementary Figure 24. Sensitive analysis of studies comparing sE-selectin levels between inactive and active IBD patients.

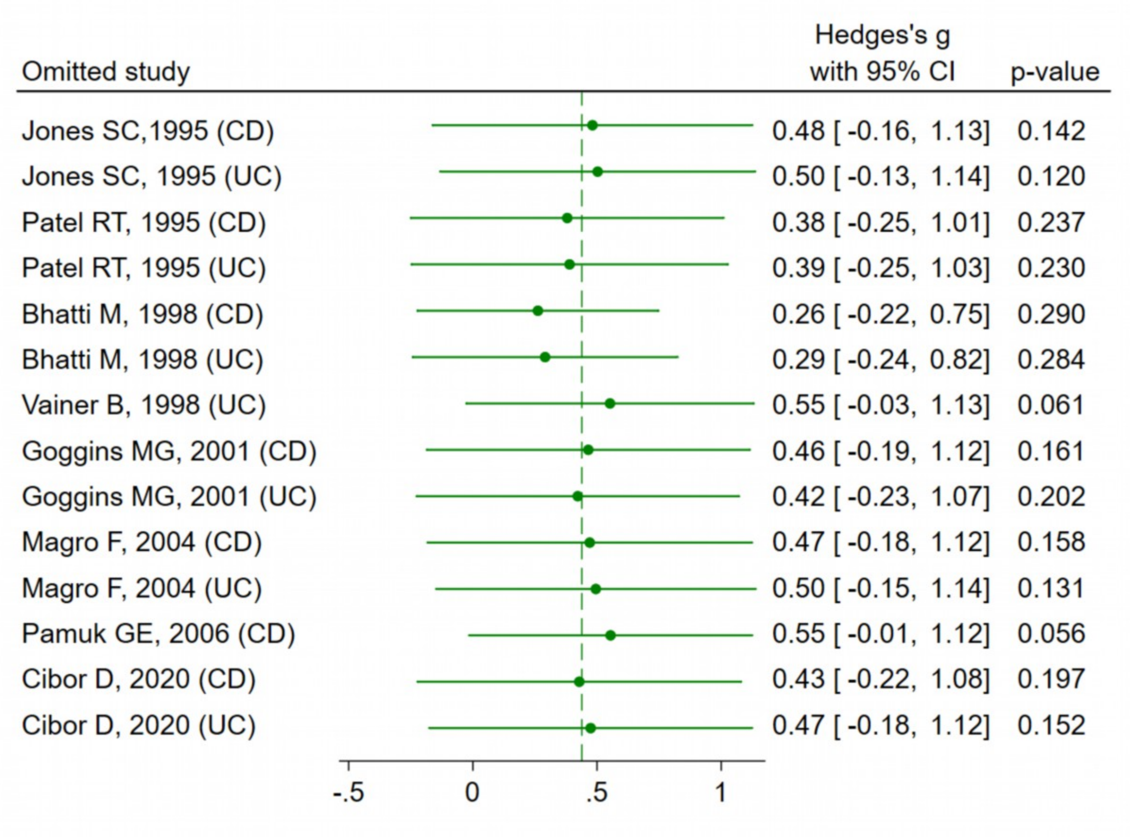

2.3.9 Supplementary Figure 25. Funnel plot of studies comparing sE-selectin levels between inactive and active IBD patients.

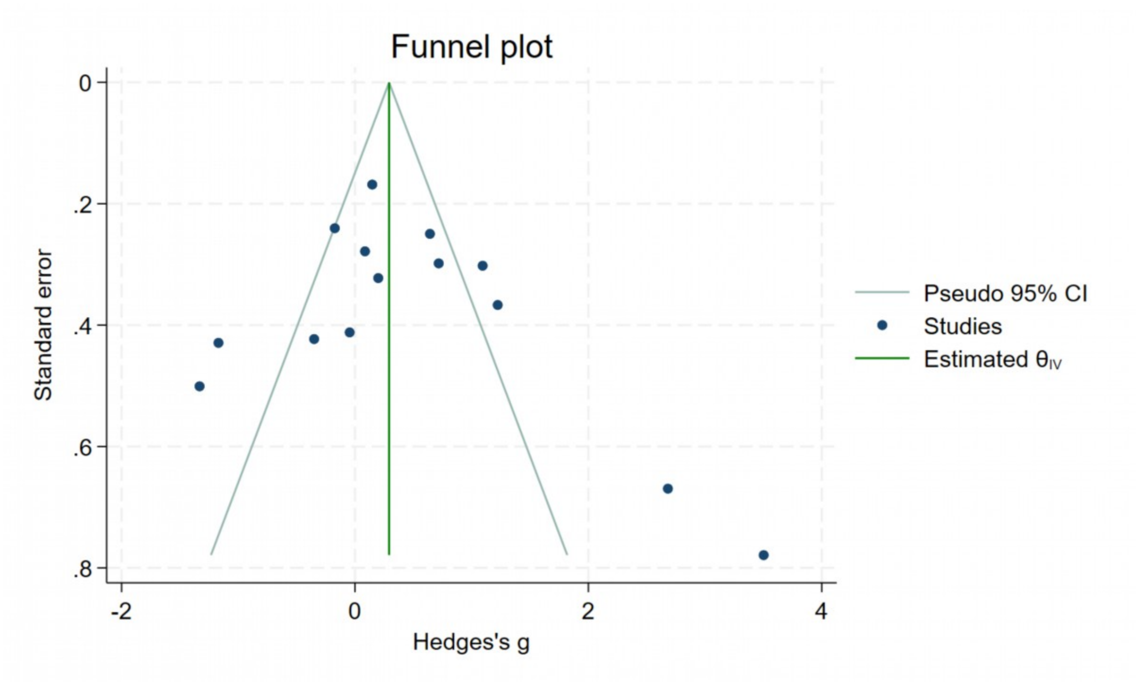

2.3.10 Supplementary Figure 26. Forest plot of studies comparing sE-selectin levels between inactive and active IBD patients, with subgroup analysis for IBD.

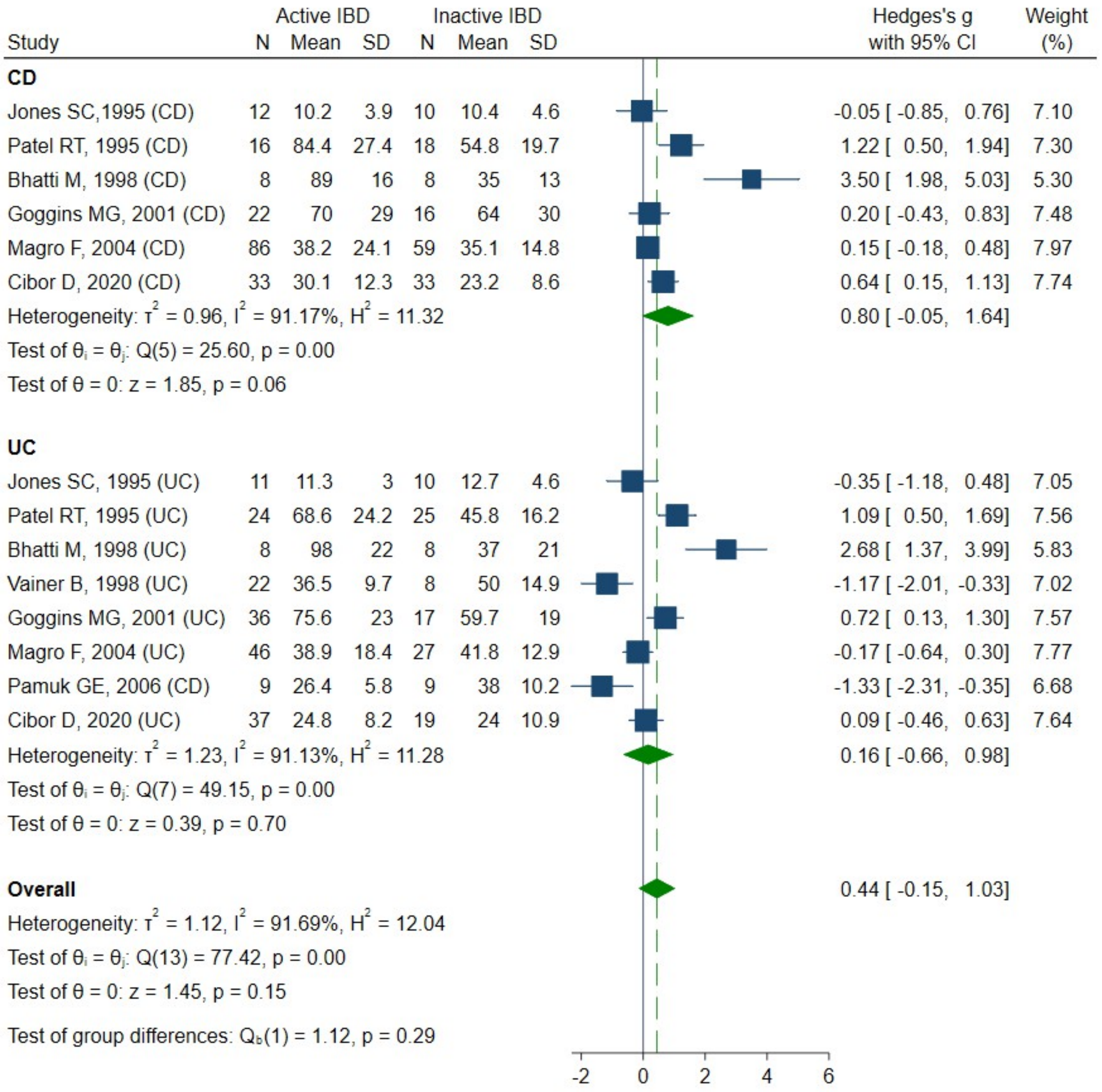

2.4 Supplementary Figures for sP-selectin.

2.4.1 Supplementary Figure 27. Forest plot of studies comparing sP-selectin levels between IBD patients and healthy controls.

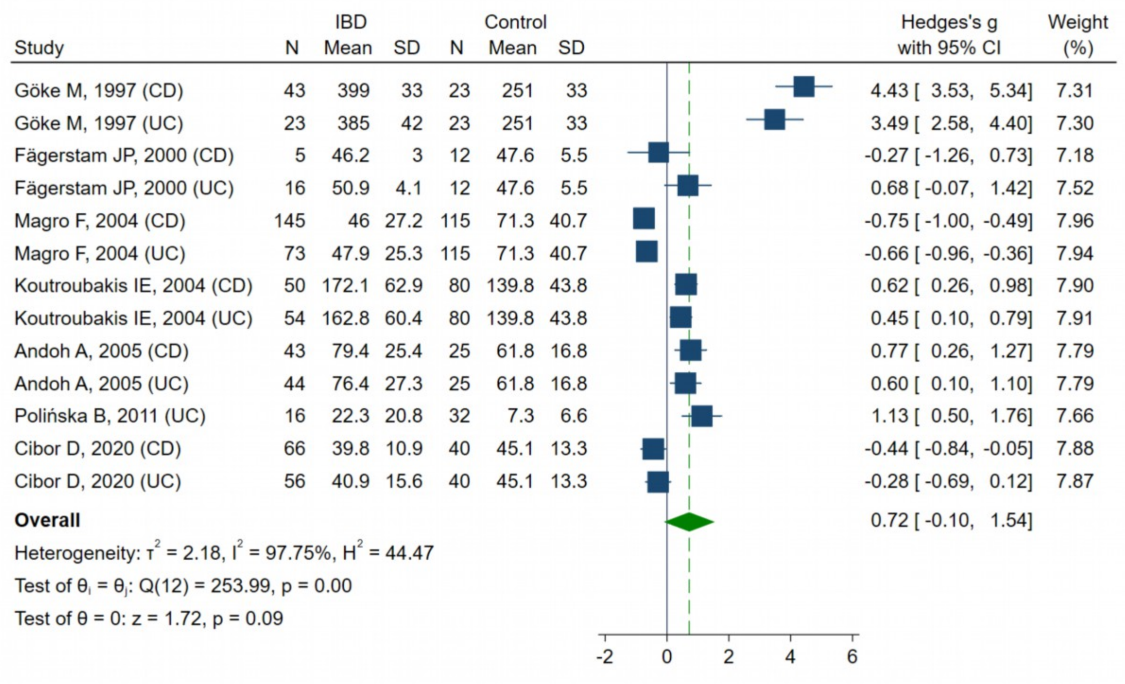

2.4.2 Supplementary Figure 28. Sensitive analysis of studies comparing sP-selectin levels between IBD patients and healthy controls.

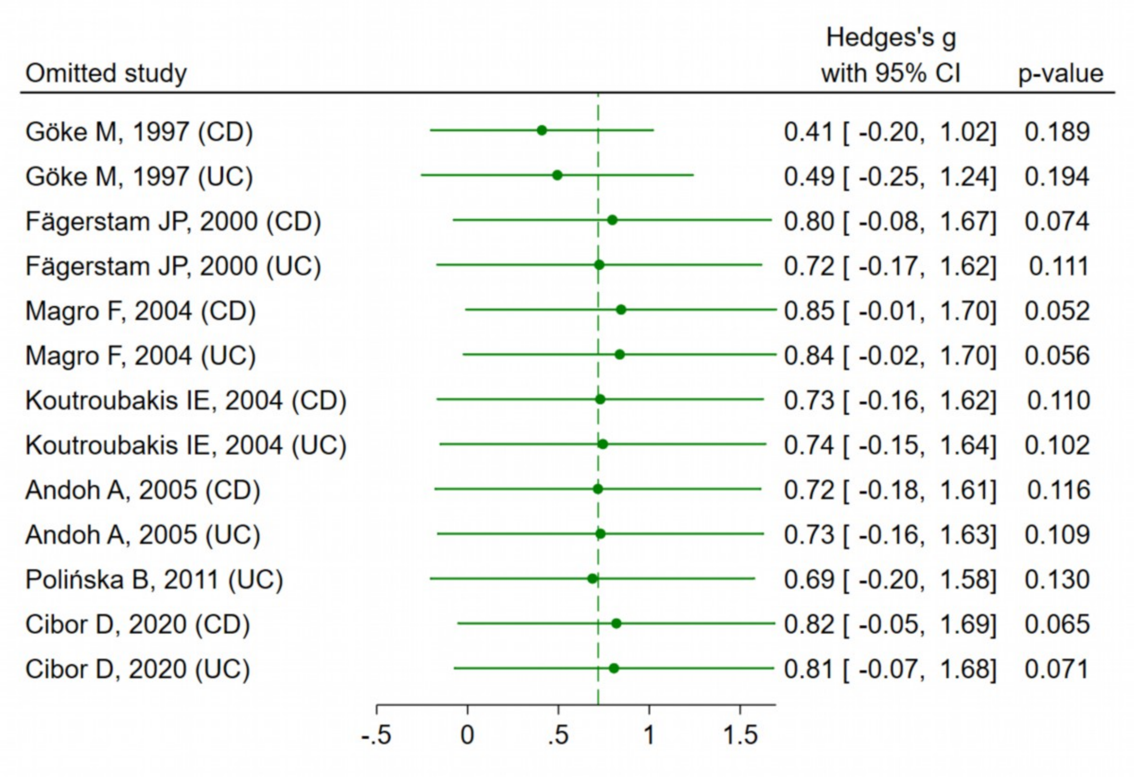

2.4.3 Supplementary Figure 29. Funnel plot of studies comparing sP-selectin levels between IBD patients and healthy controls.

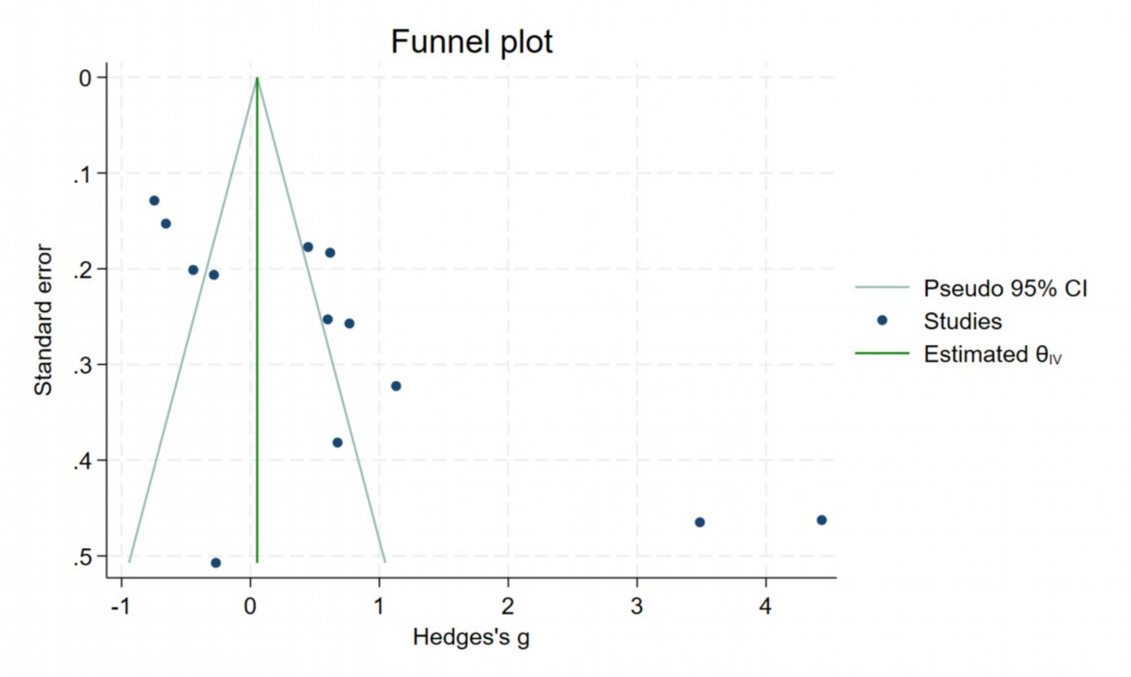

2.4.4 Supplementary Figure 30. Forest plot of studies comparing sP-selestin levels between CD and UC patients.

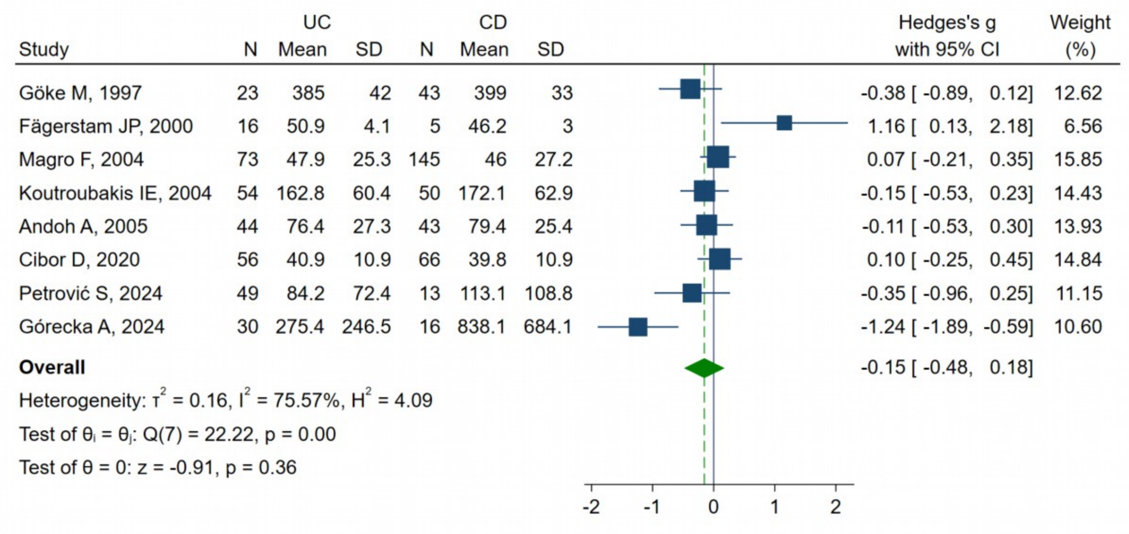

2.4.5 Supplementary Figure 31. Sensitive analysis of studies comparing sP-selectin levels between CD and UC patients.

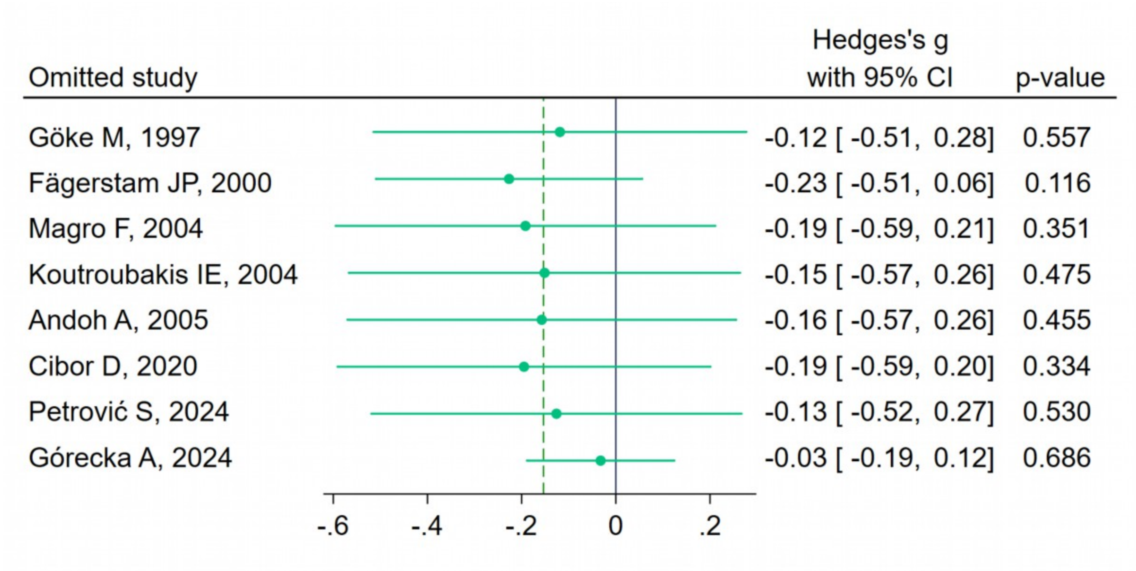

2.4.6 Supplementary Figure 32. Forest plot of studies comparing sP-selectin levels between inactive and active IBD patients.

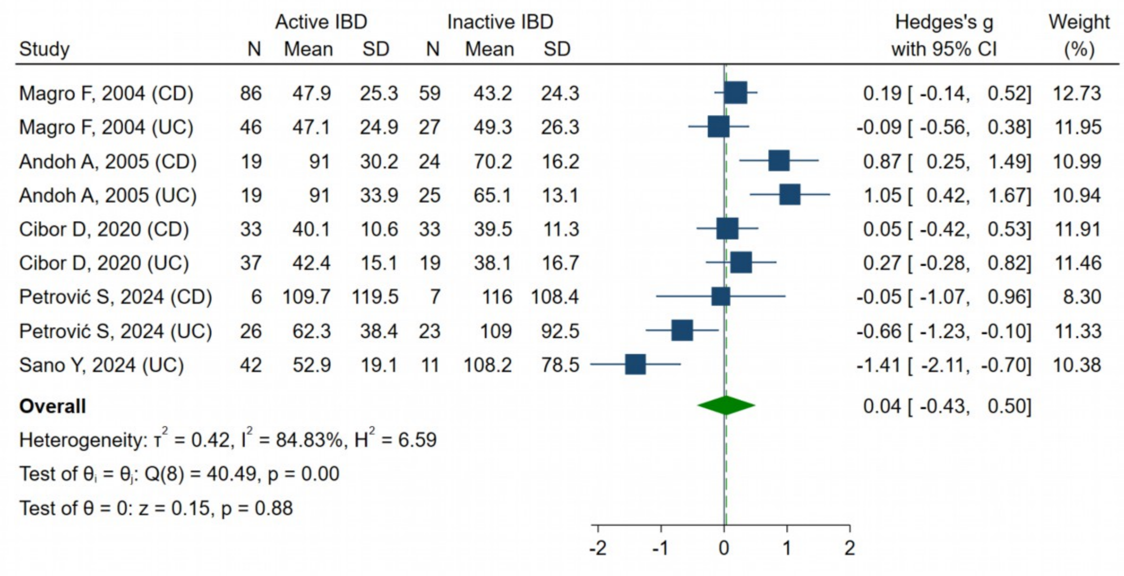

2.4.7 Supplementary Figure 33. Sensitive analysis of studies comparing sP-selectin levels between inactive and active IBD patients.

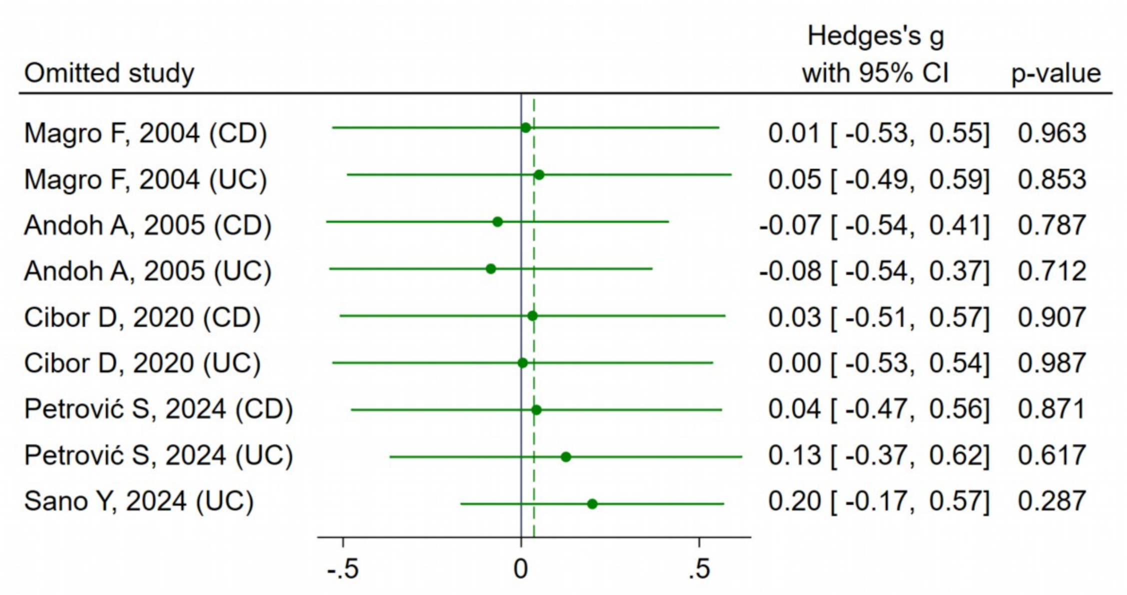

2.4.8 Supplementary Figure 34. Forest plot of studies comparing sP-selectin levels between inactive and active IBD patients, with subgroup analysis for IBD.

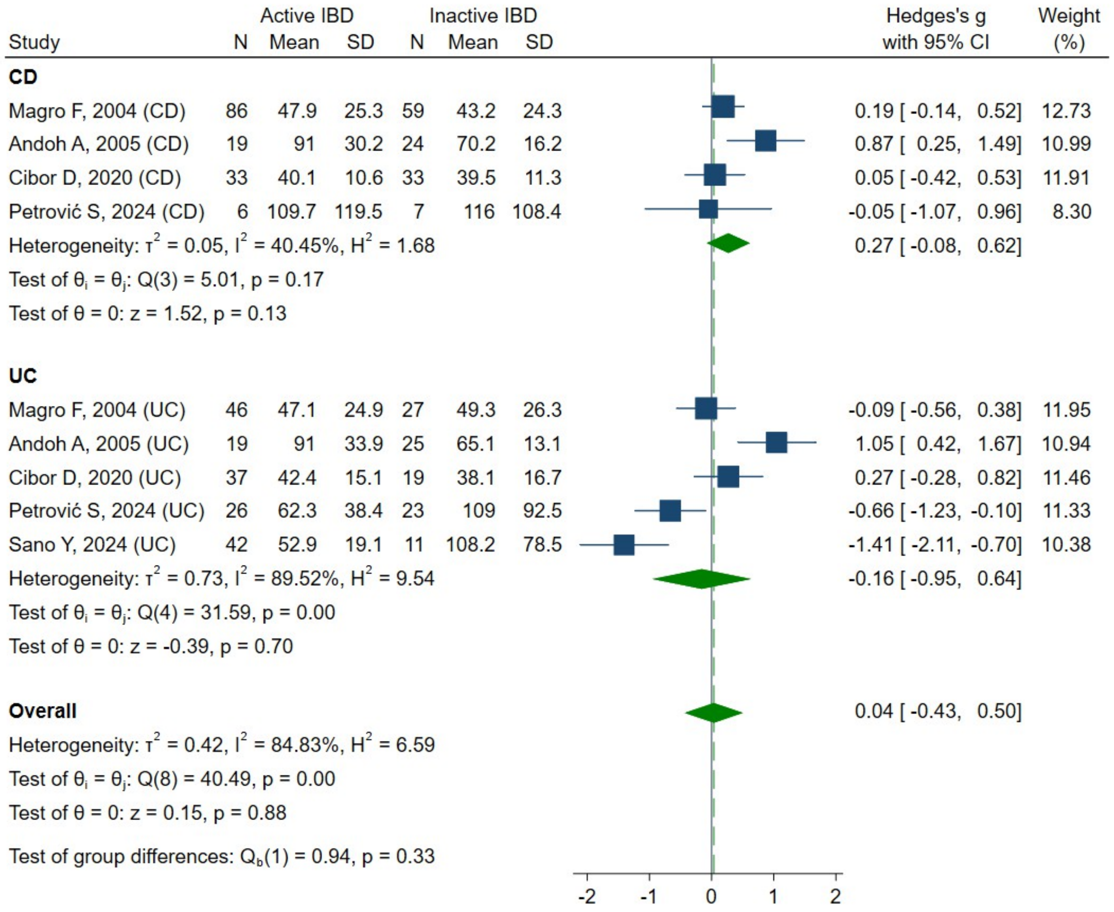

Supplement: Supplementary file 1 [file DataSheet1.pdf]
